# Examining Prenylated Xanthones as Potential Inhibitors Against Ketohexokinase C Isoform for the Treatment of Fructose-Driven Metabolic Disorders: An Integrated Computational Approach

**DOI:** 10.3390/ph18010126

**Published:** 2025-01-18

**Authors:** Tilal Elsaman, Magdi Awadalla Mohamed

**Affiliations:** Department of Pharmaceutical Chemistry, College of Pharmacy, Jouf University, Sakaka 72388, Saudi Arabia

**Keywords:** xanthones, fructose, ketohexokinase C, obesity, diabetes, dyslipidemia

## Abstract

**Background/Objectives:** Fructose-driven metabolic disorders, such as obesity, non-alcoholic fatty liver disease (NAFLD), dyslipidemia, and type 2 diabetes, are significant global health challenges. Ketohexokinase C (KHK-C), a key enzyme in fructose metabolism, is a promising therapeutic target. α-Mangostin, a naturally occurring prenylated xanthone, has been identified as an effective KHK-C inhibitor, prompting exploration of its analogs for enhanced efficacy. This study aimed to identify α-Mangostin analogs with improved inhibitory properties against KHK-C to address these disorders. **Methods:** A library of 1383 analogs was compiled from chemical databases and the literature. Molecular docking, binding free energy calculations, pharmacokinetic assessments, molecular dynamics simulations, and quantum mechani–cal analyses were used to screen and evaluate the compounds. α-Mangostin’s binding affinity (37.34 kcal/mol) served as the benchmark. **Results:** Sixteen analogs demonstrated binding affinities superior to α-Mangostin (from −45.51 to −61.3 kcal/mol), LY-3522348 (−45.36 kcal/mol), and reported marine-derived inhibitors (from −22.74 to −51.83 kcal/mol). Hits **7**, **8**, **9**, **13**, and **15** not only surpassed these benchmarks in binding affinity, but also exhibited superior pharmacokinetic properties compared to α-Mangostin, LY-3522348, and marine-derived inhibitors, indicating strong in vivo potential. Among these, hit **8** emerged as the best performer, achieving a binding free energy of −61.30 kcal/mol, 100% predicted oral absorption, enhanced metabolic stability, and stable molecular dynamics. **Conclusions:** Hit **8** emerged as the most promising candidate due to its superior binding affinity, favorable pharmacokinetics, and stable interactions with KHK-C. These findings highlight its potential for treating fructose-driven metabolic disorders, warranting further experimental validation.

## 1. Introduction

**Figure 1:**
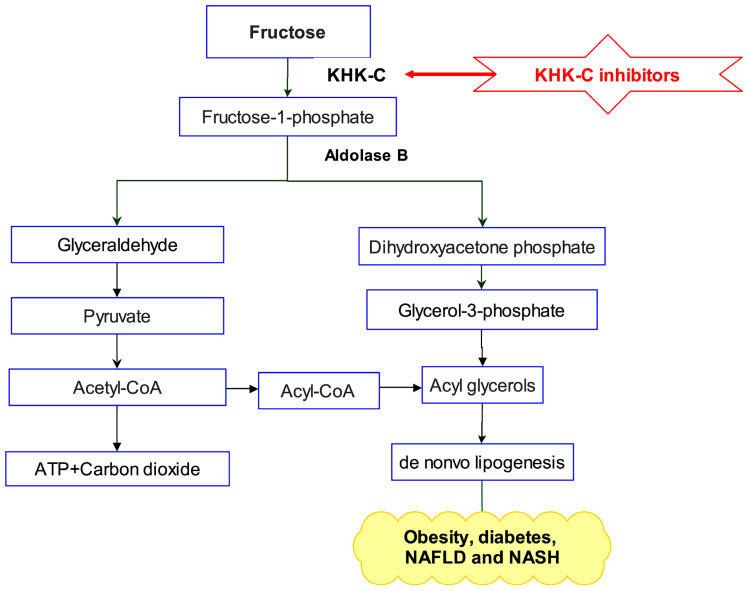
Hepatic metabolism of fructose begins with its phosphorylation by the enzyme KHK-C, producing fructose-1-phosphate. This intermediate is subsequently cleaved by aldolase B to yield the trioses glyceraldehyde and dihydroxyacetone phosphate. Glyceraldehyde is then phosphorylated to form glyceraldehyde-3-phosphate. At this stage, the phosphorylated trioses enter the glycolytic pathway and are ultimately converted into triglycerides and VLDL particles, thereby promoting lipogenesis.

**Figure 2:**
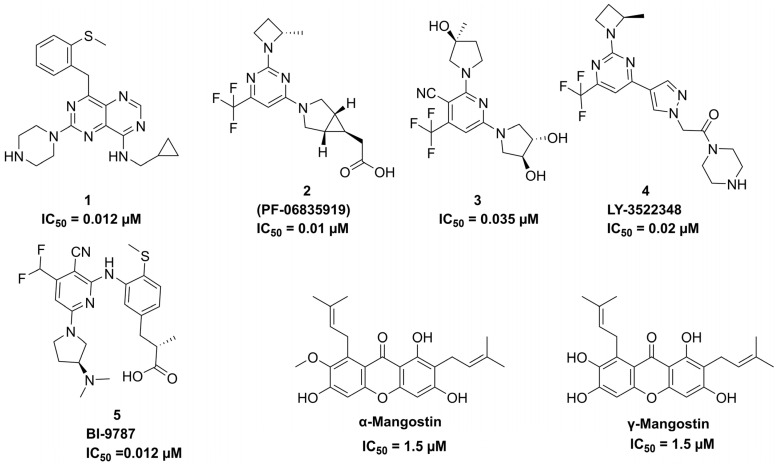
Chemical structures and IC_50_ values of reported KHK-C inhibitors.

The excessive consumption of fructose-rich sugary diets is associated with the onset of various metabolic disorders by contributing to insulin resistance and stimulating de novo lipogenesis [1,2,3,4]. Such disorders comprise obesity, diabetes, non-alcoholic fatty liver disease (NAFLD), and non-alcoholic steatohepatitis (NASH), which significantly decrease life expectancy worldwide and adversely affect quality of life [5,6]. The World Health Organization (WHO) and American Heart Association (AHA) recommend limiting daily sugar intake to below 5%. However, implementing this guideline on a global scale remains a significant challenge due to cultural dietary habits, the widespread availability of sugary foods, and varying levels of public health awareness [6,7,8]. Recent studies indicate that negative metabolic adaptations in humans are more strongly associated with excessive fructose consumption than with excessive glucose intake [9,10,11]. Furthermore, studies involving the restriction of fructose consumption in children have demonstrated improved insulin sensitivity, reduced hepatic steatosis, and a decrease in cardiovascular risk factors [6,12,13,14]. These findings further support the critical role of excessive fructose intake in driving metabolic diseases in humans. Upon oral absorption, fructose is rapidly metabolized in the liver, where the enzyme ketohexokinase (KHK) converts it into fructose-1-phosphate, utilizing adenosine triphosphate (ATP) as a cofactor (Figure 1) [15,16]. In contrast to glucose liver metabolism, which is tightly regulated, fructose is rapidly metabolized in the liver by KHK without any feedback inhibition [17]. KHK exists in two isoforms, KHK-A and KHK-C, with KHK-C being the primary enzyme for fructose metabolism in the liver, as evidenced by its lower *K_M_* and higher *V_max_* values [18]. Research demonstrated that KHK-null mice were protected from fructose-driven metabolic abnormalities, including hyperlipidemia, insulin resistance, and weight gain [15,19]. In this context, data from human genetics and nonclinical studies suggest that inhibiting KHK may serve as a promising therapeutic approach for managing fructose-driven metabolic disorders [20]. A review of the literature identified three classes of small-molecule inhibitors (Figure 2). Among these, compound **1** emerged as the most potent KHK inhibitor reported to date. However, rat pharmacokinetic studies demonstrated low exposure levels, attributed to high metabolic clearance [21]. Compound **2** (PF-06835919), developed by Pfizer through the optimization of lead compound **3**, emerged as the most promising candidate in phase II clinical trials for the treatment of NAFLD [21]. Studies have demonstrated that treatment with this agent in human patients resulted in a pharmacodynamic response, a reduction in liver fat, and an improved insulin sensitivity [6]. Another promising clinical candidate for KHK inhibition is compound **4** (LY-3522348) (Figure 2), reported by Eli Lilly and Company in 2020, which demonstrated a strong potency [22,23]. Further, Heine et al. recently reported the discovery of compound **5** (BI-9787), a potent zwitterionic KHK inhibitor notable for its high permeability and favorable oral pharmacokinetic profile in rats [24]. Furthermore, an in silico approach was adopted by Alturki to identify potent KHK-C inhibitors from natural marine organisms [5]. Another study investigating natural products as KHK-C inhibitors revealed that certain extracts and pure natural compounds exhibited potent inhibitory effects on KHK-C [25]. Given the substantial market demand, the discovery of novel KHK inhibitors with enhanced therapeutic properties is a critical area of research, as such inhibitors could effectively manage excess carbohydrates metabolism without posing mechanism-related safety risks [21,26]. Nature-derived products and their structural analogs have long served as a major source of effective therapeutics for numerous diseases [27,28]. Prenylated xanthones, secondary metabolites derived from higher plants, exhibit a wide range of biological activities, including antioxidant [29], hepatoprotective [30], anti-inflammatory [31], anticancer [32], antimalarial [33], and antibacterial effects [34,35]. α- and γ-Mangostins (Figure 2), the major prenylated xanthones of *Garcinia mangoatana* L. and some other Garcinia plants [36], have demonstrated interesting KHK-C inhibitory activity [25].

Computational approaches are being increasingly applied in the drug discovery process because they offer powerful tools for predicting the therapeutic potential of new drug candidates while reducing time and resource expenditures [37]. In this context, the objective of this study was to virtually screen a collection of 1383 α- and γ-Mangostin analogs sourced from the COCONUT database [38], PubChem [39], and the relevant published literature [36,40,41,42,43]. This screening aimed to identify xanthones with enhanced drug distribution in target tissues, improved pharmacokinetics, and potent inhibitory activity against the KHK-C enzyme, a key regulator in fructose metabolism. The identified xanthones are anticipated to pave the way for the better management and treatment of various metabolic disorders arising from excessive fructose metabolism.

## 2. Results and Discussion

**Figure 3:**
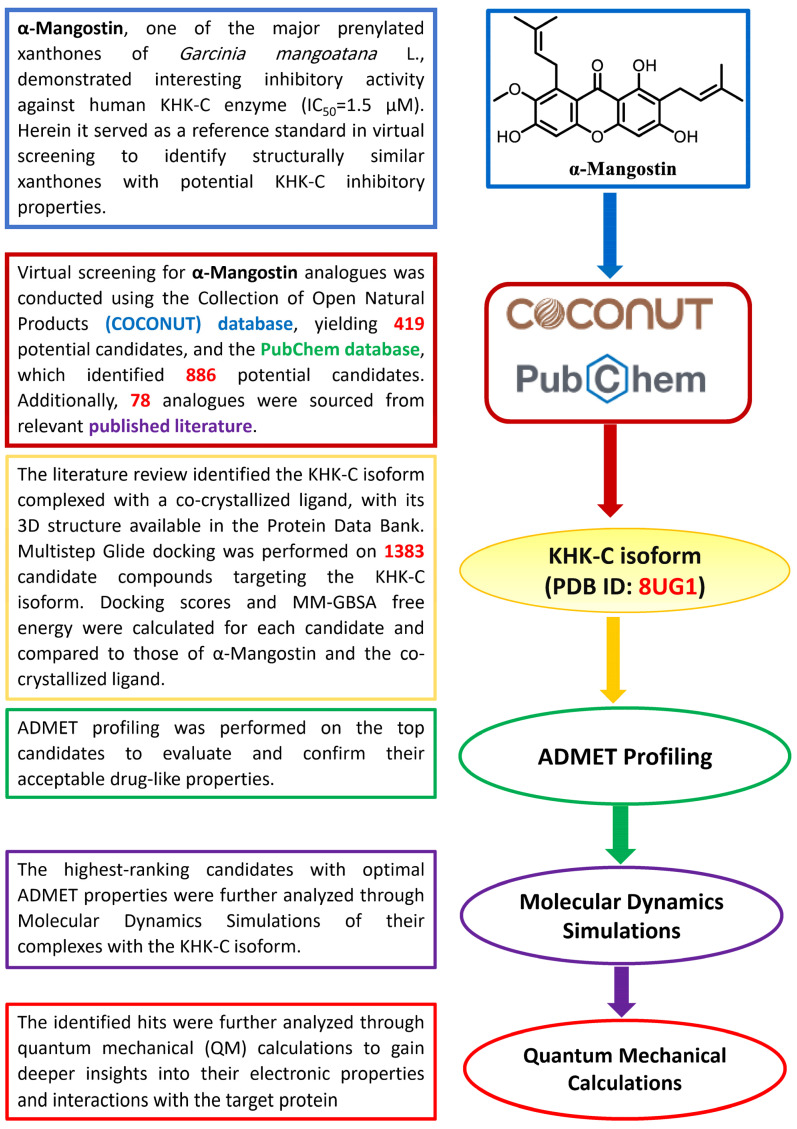
Virtual screening pipeline for the identification of prenylated xanthones as potential KHK-C inhibitors.

Clinical and preclinical studies have demonstrated that inhibiting KHK-C or implementing fructose restriction can positively impact metabolic disorders, liver health, and kidney function [6,44,45,46]. Consequently, KHK-C inhibitors hold potential as therapeutic agents for managing diabetes, obesity, NAFLD, NASH, chronic kidney disease (CKD), and diabetic kidney disease (DKD) [23,47]. In this context, integrated in silico strategies (Figure 3) were employed in this study to identify potential KHK-C inhibitors through the exploration of a pool of prenylated xanthones, which are structurally related to α- and γ-Mangostin.

### 2.1. Target Selection and Validation

A literature search identified multiple crystal structures in the Protein Data Bank (PDB), with **8UG1** [23] (1.99 Å), **8OMJ** (1.98 Å) [24], and **9FHD** (1.85 Å) [24] being selected for docking evaluation based on their high resolution. However, **9FHD** was unavailable for download, leaving **8UG1** and **8OMJ** for comparison. A protein reliability analysis favored **8UG1** due to superior R-values (R-Free: 0.205, R-Work: 0.173) and robust structural features, including excellent binding site packing and minimized steric clashes. In contrast, **8OMJ** showed higher R-Free (0.252) and significant issues, including peptide planarity and buried unsatisfied donors, which may impact docking accuracy. Thus, **8UG1** was deemed the most suitable structure for docking.

### 2.2. Molecular Docking

**Figure 4:**
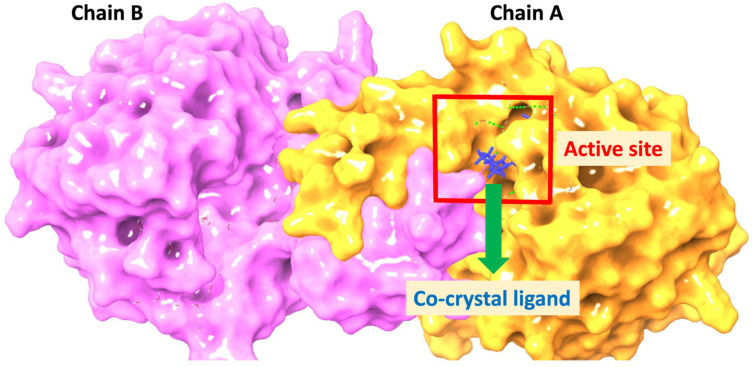
Binding mode of the co-crystal ligand in the dimeric active site of KHK-C.

In recent years, molecular docking has emerged as a critical tool and one of the most extensively utilized methods in drug discovery and development. This technique involves predicting the interactions between a small molecule and a specific protein at the molecular level [48,49]. The active site of the KHK-C protein, shown in Figure 4, is part of a dimer in the X-ray crystal structure. The co-crystal ligand occupies the ATP site of the enzyme, primarily binding to residues in chain A and interacting with the key residue Asp27 in chain B. Docking was, therefore, performed for the dimer.

**Figure 5:**
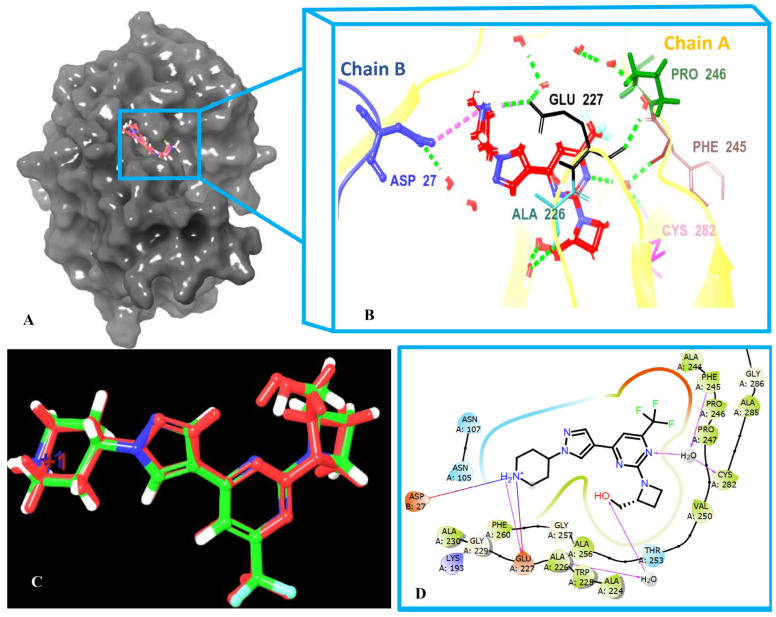
(**A**) The co-crystal ligand is positioned in the active site of KHK-C (PDB ID: **8UG1**). (**B**) Three-dimensional interactions of the co-crystal ligand with key residues, displayed in their three-letter codes. Hydrogen bonds and water bridges are shown as green dotted lines, while salt bridges are indicated by pink dotted lines. (**C**) A visual representation of the redocking process, where the original pose of the ligand is shown in green, and the redocked pose is depicted in red. (**D**) Two-dimensional interactions, with hydrogen bonds highlighted in magenta and salt bridges illustrated as a gradient line from purple to red, where purple represents the positive center and red indicates the negative center.

Before virtual screening, the co-crystal ligand was redocked three times to validate the docking efficiency (Figure 5). The RMSD between the docked and original poses was calculated and found to be below 0.5 Å, indicating the high reliability of the docking protocol. The co-crystal ligand maintained key interactions with the active site residues, as summarized in Table 1 and illustrated in Figure 5. It displayed a docking score of −9.371 kcal/mol, highlighting its strong binding affinity. The co-crystal ligand formed several key interactions with residues within the ATP binding pocket. This pocket is relatively flat, allowing the pyrazole pyrimidine core to occupy the adenine binding position [23,24]. These interactions include salt bridges with Asp27 (4.1 Å) (from subunit B) and Glu227 (2.21 Å) and water-mediated hydrogen bonds with Ala226 (2.15 and 2.19 Å), Phe245 (2.05 and 1.91 Å), and Cys282 (2.05 and 1.78 Å), as well as a direct hydrogen bond with Glu227 (3.1 Å). Furthermore, the trifluromethyl group attached to C-6 of the pyrimidine ring was within the range of the hydrophobic interactions with the proline wall (**Pro245** to **Pro247**). These interactions were similar to the reported inhibitors [20,21,24], which also exhibited strong binding affinities by forming salt bridges and hydrogen bonds with key residues in the active site, further validating the reliability of the docking approach and the relevance of the co-crystal ligand as a benchmark for identifying potential inhibitors. On the other hand, the 2-hydroxymethylazetidinyl ring system attached to C2 of the pyrimidine ring, occupied the ATP-ribose pocket, and displayed hydrophobic interactions with key residues, including Ala224, Trp225, Ala226, and Phe260. Additionally, it formed a water bridge through the hydroxyl group (OH) with a conserved water molecule, interacting with Ala226, further enhancing the binding affinity within the active site. In addition, docking of the experimentally validated compounds γ-Mangostin, α-Mangostin [25], and the clinical candidate LY-3522348 [23] against the co-crystal ligand was performed to compare their docking scores and interaction profiles with the hits identified from virtual screening. Notably, the virtual screening hits were analogs of the mangostins, making this comparison particularly significant for evaluating their potential as inhibitors of KHK-C. γ-Mangostin and α-Mangostin exhibited docking scores of −7.644 and −5.992 kcal/mol, respectively. The co-crystal ligand had a docking score of −9.371 kcal/mol, while LY-3522348 scored −8.409 kcal/mol (Table 1), indicating a slightly lower docking efficiency. LY-3522348 formed direct hydrogen bond interactions with Glu227 (1.91 Å), similar to the co-crystallized ligand, and an additional hydrogen bond with Thr253 (2.16 Å). It also exhibited a water bridge with Cys282 and Phe245, consistent with the co-crystallized ligand, but did not form a water bridge with Ala226. Furthermore, a salt bridge was observed with Glu227, as seen with the co-crystal ligand, but not with Asp27 (Table 1, Supplementary Figure S1). γ-Mangostin formed a hydrogen bond with Ala226 (1.80 Å) and exhibited water bridge interactions with Asp27, Phe245, and Cys282. Water bridge formation with Cys282 and Phe245 has been reported for several inhibitors and is considered to be essential for critical binding [20,21,23,24]. In contrast, α-Mangostin formed a hydrogen bond with Glu173 and showed water bridge interactions with Lys193 and Ala226 (Table 1, Supplementary Figure S1). Lys193 and Ala226 have been reported in the literature to interact with potent inhibitors, highlighting their relevance in binding affinity [23]. While γ-Mangostin showed a moderate similarity to the co-crystal ligand in terms of hydrogen bonds and water bridges, its overall docking score and lack of key interactions like Thr253 and salt bridges made it less favorable than LY-3522348. α-Mangostin had weaker binding interactions and diverged significantly from the co-crystalized ligand’s binding profile, suggesting a lower binding affinity and efficacy. LY-3522348 emerged as the most promising candidate, closely replicating the binding interactions of the co-crystal ligand and showing a stronger binding affinity (Table 1, Supplementary Figure S1). LY-3522348, like the co-crystal ligand and the known inhibitor PF-06835919 [21], engaged in hydrophobic interactions with the proline loop (Pro245 to Pro247) via its trifluoromethyl group. In contrast, the mangostins interacted through the prenyl unit at position 8. Furthermore, PF-06835919 formed ionic interactions with Arg108, which was critical for maintaining its potency, as reported by Zhu et al. and Heine et al. [21,24]. They hypothesized that this interaction was essential for potency. In contrast, LY-3522348, the co-crystal ligand, and the mangostins showed no interactions with Arg108. However, these compounds exhibited similar water bridge interactions with Cys282 and Phe245. Furthermore, PF-06835919 formed hydrogen bonds with Gly255 and Gly257, interactions not observed with LY-3522348, the co-crystal ligand, or the mangostins. Gly255 and Gly257 have been reported to interact with inhibitors, emphasizing their role in the binding affinity of potent compounds [21,24].

**Figure 6:**
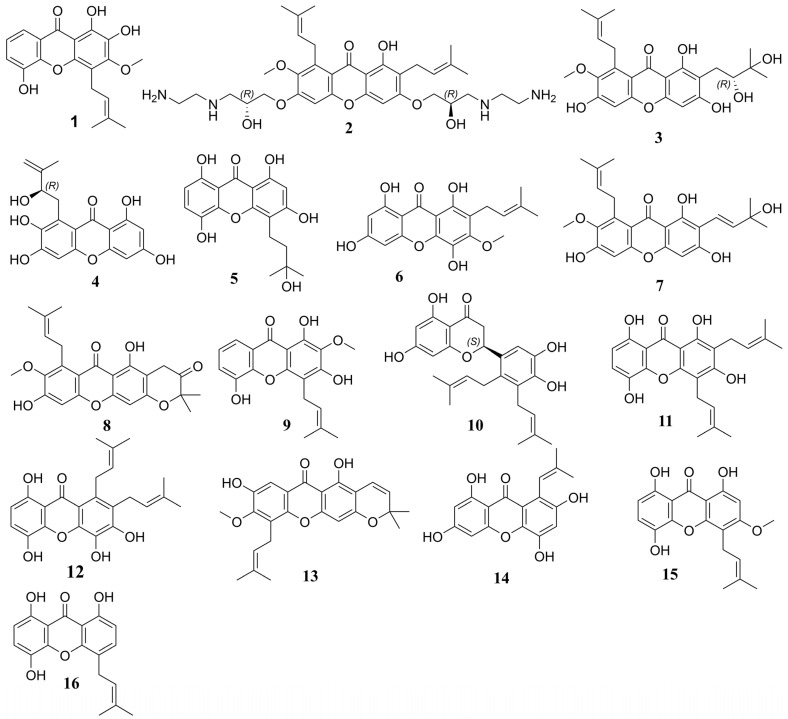
Chemical structures of leading compounds identified in virtual screening.

**Figure 7:**
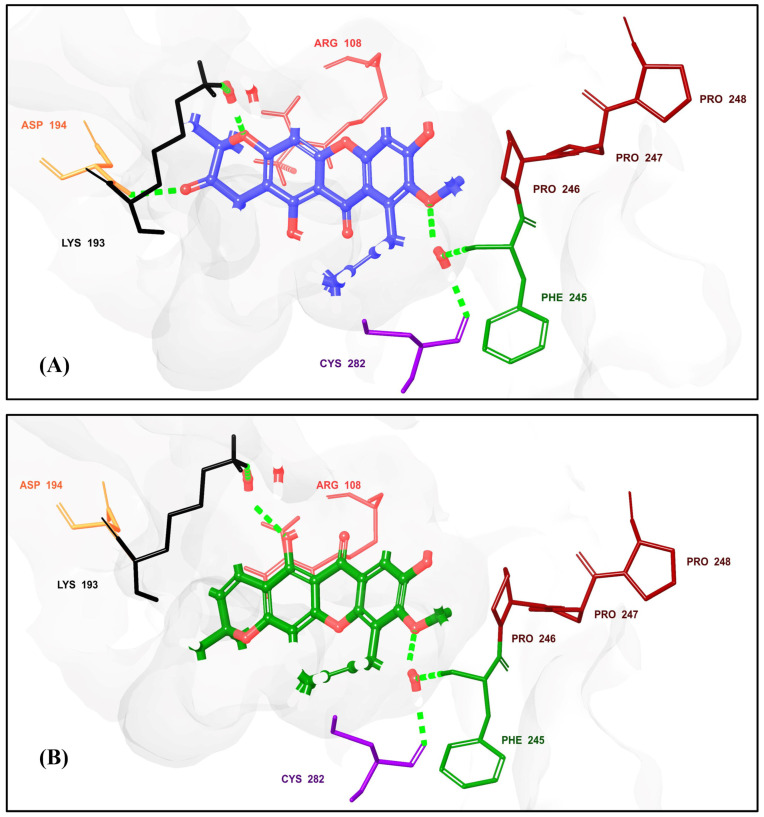
Three-dimensional interaction diagrams of two prenylated xanthones, hit **8** (**A**) and hit **13** (**B**), with KHK-C (PDB ID: **8UG1**): key residue interactions in the binding site highlighting H-bonds and water bridges (green) and Pi–cation interactions (red).

A multistep molecular docking approach was performed for 1383 prenylated xanthones, resulting in 50 compounds exhibiting docking scores higher than γ-Mangostin and α-Mangostin. Notably, 8 of these compounds displayed docking scores higher than the co-crystal ligand (from −9.380 to −10.791 kcal/mol), and 12 surpassed the clinical candidate LY-3522348, highlighting their potential as strong candidates for further investigation. Furthermore, we performed MM–GBSA calculations to refine our selection, as this method provides a more accurate estimation of binding free energies compared to docking [50]. This was applied to the 50 prenylated xanthones, ultimately affording 16 compounds (Table 1 and Figure 6) with binding energies higher than those of γ-Mangostin and α-Mangostin (−38.58 and −37.34 kcal/mol, respectively). Interestingly, these compounds exhibited similar binding affinities, which coincided with their experimental IC_50_ [25] values, further supporting that binding affinity is a reliable indicator for rectifying hits and selecting promising candidates for further development. Importantly, hits **1**, **3**, **5**, **7–9**, **13**, and **15** displayed binding affinities ranging from −45.51 to −61.3 kcal/mol, surpassing that of the clinical candidate LY-3522348 (−45.36 kcal/mol). Notably, hit **8** (−61.3 kcal/mol) and hit **13** (−59.13 kcal/mol) exhibited particularly promising binding affinities, with hit **8** approaching the binding affinity of the co-crystal ligand (−63.37 kcal/mol). Both compounds, which belong to the tetracyclic prenylated xanthone class, featured a prenyl group that formed a six-membered cyclic ring with the 3-OH group on the xanthone core. This structural similarity likely contributed to their comparable binding affinities, highlighting their potential for further optimization and experimental validation. The binding site of the target protein is relatively flat [24], which makes it particularly suitable for accommodating inhibitors with flat core structures such as xanthones. Xanthones, characterized by their planar molecular framework [51], aligned well with the flat geometry of the binding site, which enhanced their binding efficiency and interactions with key residues. Specifically, hit **8** (Figure 7) exhibited significant interactions with the binding site, including a hydrogen bond with Asp194 (2.48 Å) and several water bridge interactions with Lys193 (1.77 and 2.43 Å), Phe245 (2.22 and 1.91 Å), and Cys282 (2.22 and 1.78 Å). Additionally, it formed a π–cation interaction with Arg108 (5.92 Å). Residues such as Cys282, Phe245, and Lys193 have been previously reported in the literature as being critical for inhibitor binding, while Arg108 plays a pivotal role in optimizing the efficacy of inhibitors [21,24]. Similarly, hit **13** (Figure 7) demonstrated effective binding through water bridge interactions with Lys193 (2.65 and 2.43 Å), Phe245 (2.12 and 1.91 Å), and Cys282 (2.12 and 1.78 Å). The involvement of these residues in both hits emphasizes their importance as interaction hotspots within the binding site, further validating the potential of xanthones as promising inhibitors. The structural complementarity between the flat binding site and the planar xanthone framework underscores the efficiency of these interactions, highlighting the suitability of xanthones as candidates for targeted inhibitor development. All hits displayed a similar interaction pattern (Table 1 and Supplementary Figure S1), with slight variations in the specific residues to which they bound. Most hits effectively interacted with the crucial residues Phe245 and Cys282, aligning with their established importance in inhibitor binding. However, exceptions were observed with hits **10**, **12**, and **16**, which did not form interactions with either Phe245 or Cys282. Specific interactions were observed for individual hits, highlighting their binding potential. For example, hits **8** and **10** exhibited π–cation interactions with Arg108 at distances of 5.92 Å and 4.45 Å, respectively. Additionally, hit **4** demonstrated a salt bridge interaction with Arg108 (3.35 Å). Notably, the ionized polyamine derivative hit **2** engaged in multiple ionic bonds, including two with Glu29 (3.21 Å and 3.44 Å), two with Asp194 (4.71 Å and 3.26 Å), and one with Glu227 (4.34 Å), emphasizing its strong electrostatic interactions within the binding site. This deviation highlights the variability in the binding behavior among the hits and emphasizes the critical role of these residues in determining the binding efficiency and potential inhibitory activity of the compounds.

**Figure 8:**
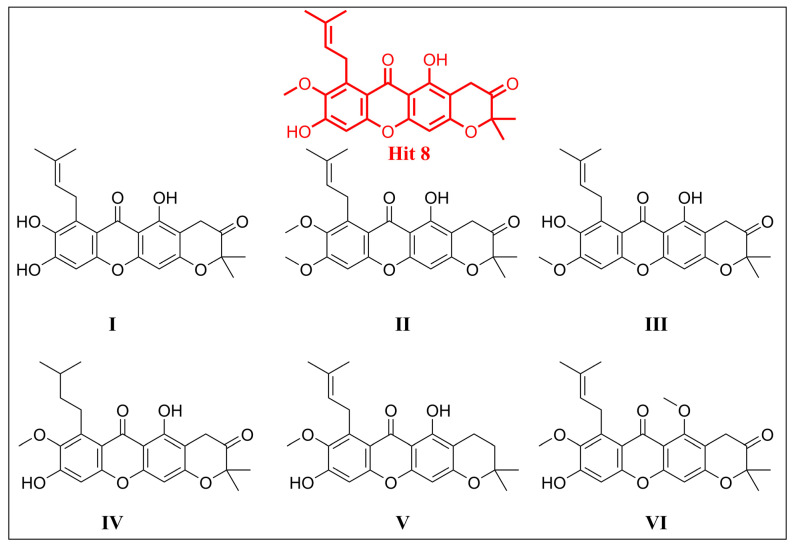

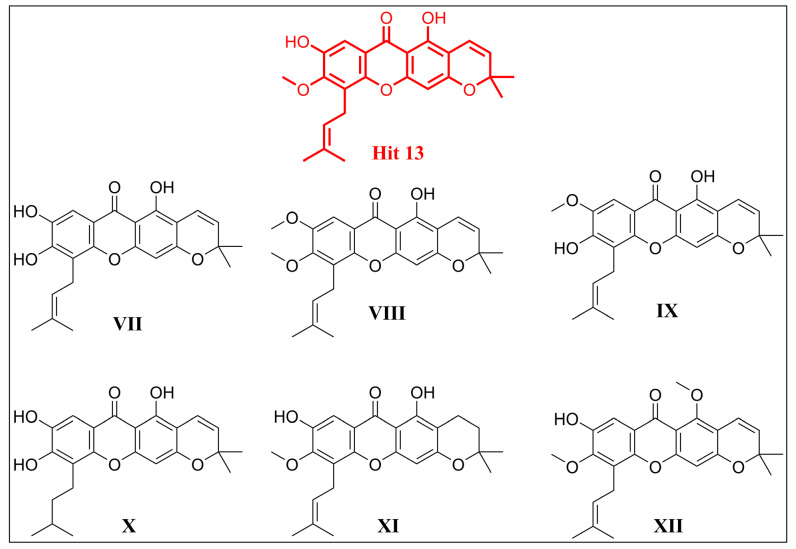
The designed analogs of hit **8** (**I–VI**) and hit **13** (**VII–XII**).

Moreover, a structure–affinity relationship study analysis was conducted for the top hits, **8** and **13**, to gain insightful information into how their structural features influenced their binding affinities, which further guided the optimization of these compounds as potential inhibitors. In this regard, six analogs for hit **8** and six analogs for hit **13** were designed (Figure 8), allowing for further exploration and optimization of the binding interactions of these compounds. All designed analogs of hit **8** (**I–VI**) demonstrated an inferior binding affinity (from −19.43 to −52.6 kcal/mol) compared to hit **8**, indicating that hit **8** possesses the essential structural features required for superior interactions with the target. In contrast, the designed analogs of hit **13**, specifically **VII** and **XI**, exhibited significantly enhanced binding affinities of −66.75 and −64.80 kcal/mol, respectively. These values surpassed both the binding affinity of the original hit **13** (−59.13 kcal/mol) and that of the co-crystal ligand (−63.37 kcal/mol), demonstrating their superior interaction potential. Structurally, analog **VII** differed from hit **13** by replacing the methoxy group at the ninth position with an additional hydroxy group, which increased its polarity and enhanced its hydrogen bonding capability. Analog **XI**, on the other hand, differed from hit **13** in that it lacked the double bond in the pyrano ring system. This structural modification likely contributed to its improved binding affinity by increasing its rigidity and stability, enhancing hydrophobic interactions, and possibly allowing for a better conformational fit within the target’s binding site. These modifications likely played a key role in the enhanced binding affinities observed for both analogs. Notably, both analogs **VII** and **XI** can be easily synthesized in one step from hit **13**, making them cost-effective and highly accessible for further development. In contrast, other analogs exhibited significantly inferior binding affinities (ranging from −21.65 to −53.38 kcal/mol), indicating that these modifications disrupted the interaction network, reducing their overall binding efficacy.

**Figure 9:**
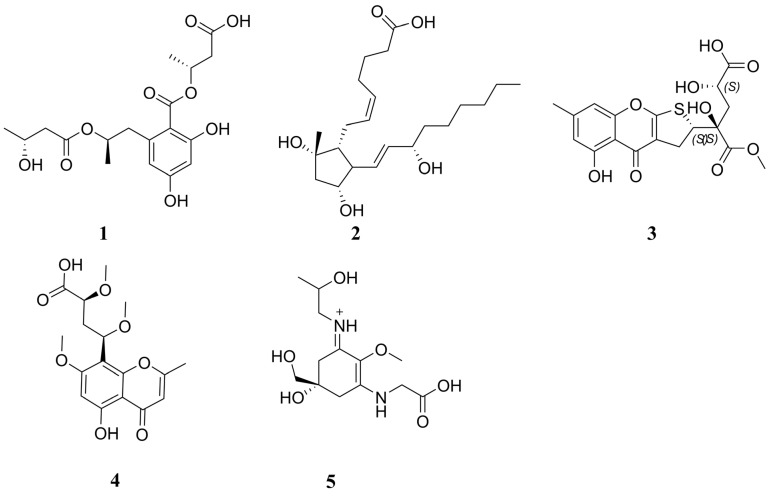
Chemical structures of the top five marine-derived natural product inhibitors identified by Alturki and reported as potential inhibitors of KHK-C.

To contextualize the findings, a comparative analysis was conducted between the top identified prenylated xanthones and the top five marine-derived natural product inhibitors (Figure 9) reported by Alturki [5]. This analysis focused on evaluating and comparing the binding affinities of these compounds with KHK-C (PDB ID: **8UG1**). As illustrated in Table 1 and Table 2, the prenylated xanthones identified in the present study, specifically hits **3**, **7**, **8**, **9**, and **13**, exhibited better binding affinities (from −53.01 to −61.30 kcal/mol) than the marine-derived natural products (from −22.74 to −51.83 kcal/mol). Notably, hits **8** (−61.30 kcal/mol) and **13** (−59.13 kcal/mol) demonstrated significantly enhanced binding affinities, further emphasizing their superior interaction potential. These findings suggest that the prenylated xanthones identified in this study are more promising candidates than the marine-derived compounds reported by Alturki [5], highlighting the superior potential of our identified hits for further development.

### 2.3. Binding Free Energy Calculations and MM–GBSA Breakdown Analysis

**Figure 10:**
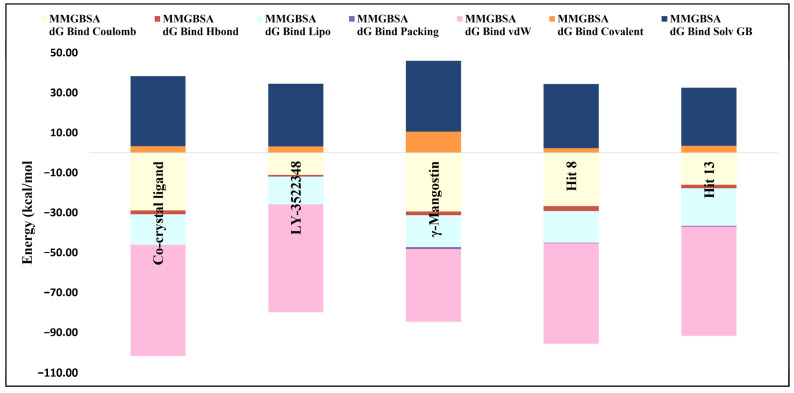
Detailed energy contributions for the co-crystal ligand, LY-3522348, γ-Mangostin, hit **8**, and hit **13**.

This section of the research introduces an analysis aimed at evaluating the binding efficiencies and interaction profiles of hit **8** and hit **13**, which were considered as the primary hits. In this context, γ-Mangostin and the co-crystal ligand, LY-3522348, were used as reference points for comparison. The goal was to identify and highlight the key factors that influenced their molecular interactions and binding performance, thereby providing insights into the potential efficacy of the compounds under investigation. The energy breakdown and binding profiles of the co-crystal ligand, LY-3522348, γ-Mangostin, hit **8**, and hit **13** (Figure 10) illustrated their varying molecular interaction efficiencies. Among the compounds, the co-crystal ligand exhibited the strongest binding energy at −63.37 kcal/mol, serving as a benchmark for comparison. Hit **8** closely followed with −61.30 kcal/mol, and hit **13** demonstrated a comparable binding efficiency at −59.13 kcal/mol. In contrast, LY-3522348 and γ-Mangostin showed significantly lower binding energies at −45.36 kcal/mol and −38.58 kcal/mol, respectively. Coulomb energy contributions (Figure 10) were prominent in γ-Mangostin (−29.44 kcal/mol) and the co-crystal ligand (−28.87 kcal/mol), underscoring the importance of polar interactions. LY-3522348 displayed the weakest Coulomb interaction at −11.12 kcal/mol, which limited its binding efficiency. Hydrophobic interactions, particularly van der Waals (vdW) forces, were major contributors to binding across all compounds (Figure 10). The co-crystal ligand (−55.77 kcal/mol) and hit **13** (−54.74 kcal/mol) led in vdW contributions, reflecting their strong hydrophobic interactions. Hit **8** followed closely at −50.24 kcal/mol, while γ-Mangostin’s reduced vdW interaction (−36.49 kcal/mol) limited its overall binding efficacy. The solvation penalty (Solv GB) consistently impacted binding, with γ-Mangostin (35.48 kcal/mol) and the co-crystal ligand (35.13 kcal/mol) showing the highest penalties. Packing energies highlighted the stability of the binding geometries, with hit **13** (−18.85 kcal/mol) and hit **8** (−15.95 kcal/mol) excelling in this aspect. Hydrogen bonding contributions were modest, but still influential in stabilizing interactions. Structurally, the co-crystal ligand’s trifluoromethyl moiety and aromatic rings facilitated strong polar and hydrophobic interactions. LY-3522348, while also featuring polar groups, fell short due to its weaker Coulomb contributions. γ-Mangostin’s xanthene scaffold, enriched with hydroxyl groups, enabled significant polar interactions, but its limited vdW contributions and higher solvation penalties hindered its binding efficiency. Hit **8** and hit **13**, both pyrano xanthene derivatives, shared similar binding profiles. Hit **8** displayed stronger vdW contributions, while hit **13** excelled in packing energy, leading to more stable binding geometries. Hit **8** and hit **13** emerged as the most promising candidates, given their robust energy profiles and efficient binding interactions. γ-Mangostin, while weaker overall, displayed potential in contexts prioritizing polar interactions. Overall, this evaluation effectively connected the energy breakdown data to the chemical structures of the top hits **8** and **13**, shedding light on their binding efficiencies and paving the way for further optimization efforts.

### 2.4. ADMET Profiling

The early evaluation of drug-like properties through in silico methods plays a pivotal role in the early phases of drug discovery [52]. These computational approaches allow for the analysis of essential characteristics such as molecular weight, solubility, membrane permeability, and toxicity profiles. By identifying favorable candidates and discarding those with less promising properties at the outset, in silico tools help to optimize the drug development pipeline, improving efficiency and increasing the chances of success [53,54]. In this context, the ADMET properties of the top 16 hits were evaluated and compared with LY-3522348 and γ-Mangostin (Supplementary Table S1). This comparison aimed to assess how the hits aligned with favorable drug-like characteristics, providing insights into their potential for further development as therapeutic candidates. An overall ADME compliance score—drug-likeness parameter (indicated by #stars) was utilized to evaluate how the compounds’ properties compared to those of established drugs. It indicates the number of property or descriptor values that fall outside the 95% confidence interval of similar values observed in known drugs [55,56]. In the present study, the #stars parameter highlighted clear differences in drug-likeness among the compounds. LY-3522348, along with most hits **(1**, **3–9**, and **13–16)**, scored zero, signifying that their property or descriptor values fell within the 95% range of similar values for known drugs, indicating excellent compliance with drug-likeness rules. γ-Mangostin and hits **10**, **11**, and **12** scored one, suggesting slight deviations from the 95% range, but still remaining within acceptable drug-likeness thresholds. Notably, hit **2**, with a score of eight, had significant deviations from the typical drug-like properties, indicating that its property or descriptor values fell well outside the 95% range of similar values for known drugs. This substantial deviation raises concerns about its potential for development as a lead compound. The physicochemical properties of a drug, including its size, solubility, and lipophilicity, are crucial parameters that significantly influence its therapeutic effectiveness [57]. The physicochemical parameters demonstrated molecular weights ranging from 312.3 (hit **16**) to 444.5 (hit **3**), all within the permissible range of 130–725, reflecting an appropriate size for drug-like molecules. The solvent-accessible surface area (SASA) values fell between 525.5 (hit **16**) and 710.4 (hit **7**), well within the permissible limits of 300–1000. These values suggest adequate interaction with aqueous environments. Compounds with a poor permeability are often associated with unfavorable ADME profiles, which can result in a reduced therapeutic efficacy [57]. The ability of a drug to permeate cell membranes is an essential factor that influences its absorption rate and extent in the human body, directly impacting the bioavailability of the compound [58]. The polar surface area (PSA) is commonly utilized in drug discovery and development due to its correlation with a compound’s capacity to cross cell membranes [59]. The PSA values (Supplementary Table S1) ranged from 86.3 (hit **13**) to 177.5 (hit **2**), within the acceptable range of 7–200, indicating that these compounds are likely to efficiently permeate biological membranes. Hydrogen bond donors (donorHBs) ranged from 1 (hits **8**, **13**, **15**, **16** and LY-3522348) to 4 (hits **3** and **4**), staying well within the permissible range of 0–6. However, hit **2** exhibited eight hydrogen bond donors, which exceeds the typical acceptable range, suggesting that it may experience challenges in terms of membrane permeability and bioavailability. Similarly, the hydrogen bond acceptor (accptHB) values (Supplementary Table S1) ranged from 2.8 (hit **16**) to 12.9 (hit **2**), also meeting the acceptable limits of 2–20. These parameters indicate a balance of hydrophilic and hydrophobic interactions, which is essential for oral bioavailability, one of the greatest advantages of small-molecule drugs, especially considering that oral administration is superior to other dosing routes [60]. In this regard, the most essential criterion for developing a drug to treat fructose-driven metabolic disorders is its suitability for oral administration, as reported for many recently disclosed candidate molecules [21,23,24]. However, even some of the disclosed potent KHK-C inhibitors have shown a low to moderate bioavailability, highlighting the need for potent and orally bioavailable drug candidates. Thus, in the present study, we evaluated the percentage of the oral bioavailability of our identified hits to assess their potential as effective drug candidates (Supplementary Table S1). Hits **8**, **13**, and **11** demonstrated the highest oral bioavailability, with hits **8** and **13** reaching 100.0% and hit **11** achieving 95.8%. These values highlight their exceptional potential as lead candidates for oral drug development, ensuring efficient absorption and therapeutic efficacy. Additionally, hits **7**, **9**, **15**, and **16** showed a high bioavailability (>90%), further supporting their promise for oral administration. In contrast, hit **2** exhibited a 0.0% bioavailability, indicating poor absorption, which could be attributed to its polarity, large size, and possible metabolic instability. Hits with a moderate bioavailability, such as **4**, **5**, and **14**, require structural or formulation improvements to enhance their absorption.

Compared to established standards such as LY-3522348 (80.35%) and γ-Mangostin (92.30%), a significant number of the hits demonstrated a superior bioavailability, indicating their potential as more effective drug candidates. Notably, when compared to a marine-derived natural product, which exhibited oral absorption rates ranging from 17.6% to 59.3% [5], the hits showed a significantly better oral absorption and bioavailability. This enhanced absorption rate is crucial for drug development, as it suggests that these hits may have a greater efficacy when administered orally, thus offering an improved therapeutic potential. The favorable oral bioavailability of these hits also positions them as promising candidates for further preclinical and clinical evaluations, especially in comparison to other compounds with suboptimal absorption profiles. Further, the absorption parameters revealed substantial variability in QPPCaco values (Supplementary Table S1). LY-3522348 scored 165.9, indicating a moderate intestinal permeability, while γ-Mangostin achieved 269.5, reflecting a slightly better absorption. Hits such as **13** (715.6) and **8** (675.4) demonstrated an excellent absorption potential, surpassing the threshold for high absorption (QPPCaco > 500). In contrast, hit **16** (417.7), hit **9** (415.5), and hit **7** (303.2) demonstrated a moderate absorption potential, which was better than LY-3522348 (165.9) and γ-Mangostin (269.5), suggesting they will support a more favorable bioavailability. Plasma protein binding plays a significant role in shaping the pharmacokinetics and pharmacodynamics of drugs. Although drugs with high plasma protein binding may have a prolonged duration of action, this also decreases the amount of free drug available, which could potentially reduce their therapeutic effectiveness [61]. QPlogKhsa analysis (Supplementary Table S1) revealed that hits **11**, **13**, and γ-Mangostin demonstrated the highest binding affinities (above 0.5). LY-3522348, hit **2**, hit **4**, and hit **14** showed poor binding (below 0), with hit **4** having the lowest value (−0.3). Moderate binding was observed in hits **1**, **3**, **5**, **6**, **8**, **9**, **15**, and **16**. The moderate to high binding affinities observed in several hits suggest that these compounds are more likely to remain in circulation longer, offering an extended duration of action. This could contribute to a better efficacy and a reduced dosing frequency, improving their potential as drug candidates. Human ether-a-go-go-related gene (hERG) channel inhibition by small molecules is a significant concern in drug development within the pharmaceutical industry [62]. QPlogHERG is a predictive parameter that was employed to evaluate the potential cardiotoxicity of the top hits by estimating their interaction with the hERG potassium channel. Hits with QPlogHERG values below −6, such as hit **2** (−9.0), were classified as being high risk for hERG channel blockade. Hits with QPlogHERG values between −5.0 and −5.6, including γ-Mangostin (−5.341) and hit **13** (−5.5), fell under the moderate risk category for potential hERG inhibition. On the other hand, hits with QPlogHERG values above −5.0, such as LY-3522348 (−4.651) and hit **1** (−4.6), indicated a low risk, suggesting minimal concerns regarding hERG channel blockade. Overall, most hits in this study were classified as being low risk, with values indicating a safer profile for drug development. Metabolic stability refers to how readily compounds undergo biotransformation, which is an important factor in selecting or designing drugs with desirable pharmacokinetic characteristics [63]. The analysis of metabolites within the permissible range of 1 to 8 showed that the identified hits exhibited a better metabolic stability than γ-Mangostin, which had 10 metabolites. LY-3522348, the clinical candidate, had only one metabolite, indicating a high stability. Hits such as hit **1**, hit **4**, hit **8,** and hit **15** showed seven metabolites, which is still within an acceptable range, indicating a good stability. Hits **5**, **13**, **14**, and **16**, with six metabolites, suggested a low metabolic instability. Hits like hit **6**, hit **7**, and hit **9** showed eight metabolites, suggesting a good stability. Hits **3**, **10**, **11**, and **12** had 10 metabolites, showing a higher metabolic instability. Hit **2** had 18 metabolites, indicating a higher metabolic instability. Overall, the identified hits showed an improved stability and were better candidates for drug development compared to γ-Mangostin. Both Lipinski’s Rule of Five (maximum violations: four) and Veber’s Rule of Three (maximum violations: three) were satisfied by all compounds, indicating that they adhered to the fundamental guidelines for drug-like properties. This suggested that the compounds had promising potential for oral bioavailability and suitable pharmacokinetic profiles, essential for their further development as therapeutic agents. The outcome of this analysis highlights the promising drug-like properties of several of the identified hits, with favorable ADMET profiles and a high oral bioavailability potential. Notably, compounds like hits **8** and **13** exhibited exceptional absorption and metabolic stability, making them strong candidates for further investigation.

### 2.5. Molecular Dynamics (MD) Simulations

**Figure 11:**
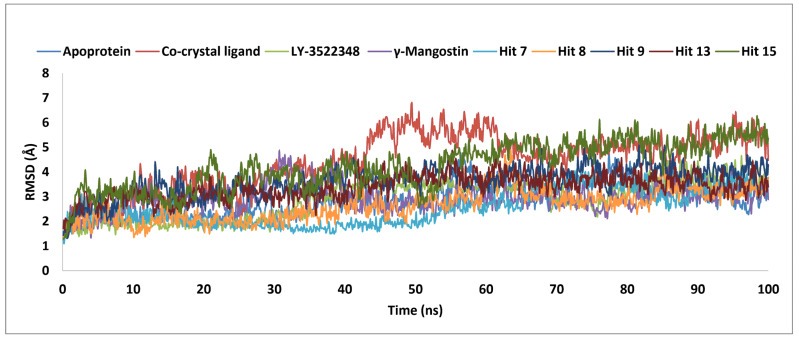
RMSD analysis of hits **7**, **8**, **9**, **13**, **15**, γ-Mangostin, LY-3522348, co-crystal ligand and apoprotein during 100 ns MD simulations.

**Figure 12:**
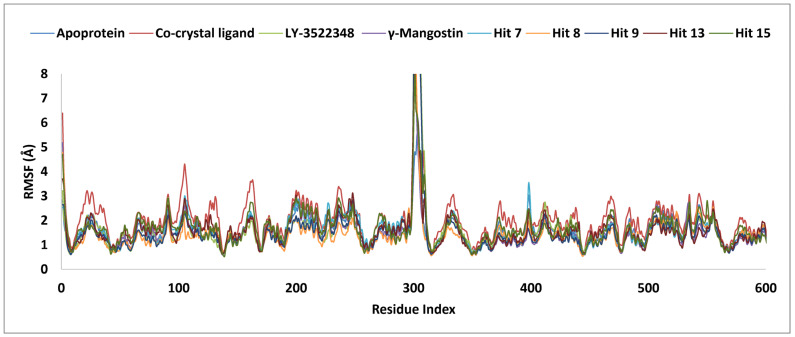
RMSF analysis of hits **7**, **8**, **9**, **13**, **15**, γ-Mangostin, LY-3522348, co-crystal ligand, and apoprotein, highlighting residue flexibility during 100 ns MD simulations.

The five hits, namely hits **7**, **8**, **9**, **13**, and **15**, with binding affinities higher than the clinical candidate LY-3522348 (<−45.36 kcal/mol), an oral bioavailability exceeding 90%, and a favorable metabolic stability, were selected for MD simulations. The aim was to gain a deeper understanding of the dynamic behavior of the selected hits at the molecular level. These simulations allowed for a detailed investigation into how these hits interacted with KHK-C, assessing the strength and nature of these interactions. Additionally, the original γ-Mangostin, LY-3522348, and the co-crystal were also included for comparative analysis to benchmark the performances and interactions of the selected hits. MD simulations have been proven to be a powerful tool in drug discovery, offering insights into the stability, flexibility, and interaction patterns of potential drug candidates under near-physiological conditions [50]. The analysis of the RMSD and RMSF values for the investigated molecules, as summarized in Table 3 and visualized in Figure 11 and Figure 12, offered valuable insights into their dynamic behavior during the MD simulations. For RMSD (Figure 11), the Apo structure exhibited an average of 2.9 Å, reflecting moderate structural deviations, while the co-crystal ligand showed a higher average RMSD of 4.4 Å, indicative of significant conformational changes relative to the reference structure. Hits **7** and **8** demonstrated lower RMSD averages of 2.6 Å and 2.7 Å, respectively, underscoring their superior structural stability in comparison to the other hits. When evaluated against the top marine-derived hits, hits **7** and **8** showcased a remarkable stability, as evidenced by their reduced RMSD values and consistently steady profiles throughout the simulation period. These findings emphasize their outstanding dynamic behavior and robust structural integrity within the binding site, surpassing the stability metrics reported for other marine-derived compounds [5] and solidifying their potential as highly promising candidates for further development. Notably, the co-crystal exhibited significant fluctuations between 40 and 70 ns, reflecting dynamic instability during this period, whereas hit **15** showed substantial fluctuations throughout the entire 100 ns simulation, indicating its relatively less stable nature. In contrast, hits **7** and **8** stabilized early in the simulation and maintained steady profiles throughout the 100 ns run, reinforcing their suitability as stable molecular interactions. The maximum RMSD values further highlighted the co-crystal ligand and hit **15** as having the highest deviations, at 6.8 Å and 6.3 Å, respectively, while hits **7** and **8** remained consistently lower, demonstrating an enhanced binding stability. The RMSF analysis (Figure 12) complemented these findings by revealing residue-level fluctuations, with the Apo structure and hits **7** and **8** maintaining consistent averages of 1.6 Å, 1.6 Å, and 1.4 Å, respectively, indicating a lower overall flexibility. LY-3522348 exhibited the highest maximum RMSF value of 14.3 Å, signifying a localized flexibility at specific regions. Residues 300–307, located in the terminal loops at the junction between subunits A and B of the KHK-C enzyme, displayed an elevated flexibility across all systems, underscoring their role as a structural hinge. This inherent mobility is critical for inter-subunit communication and may influence ligand binding dynamics.

**Figure 13:**
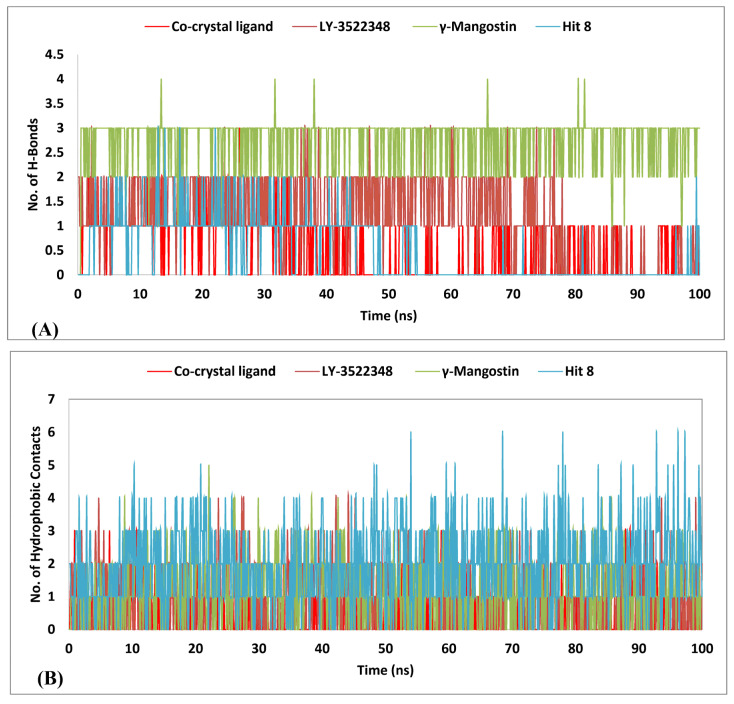

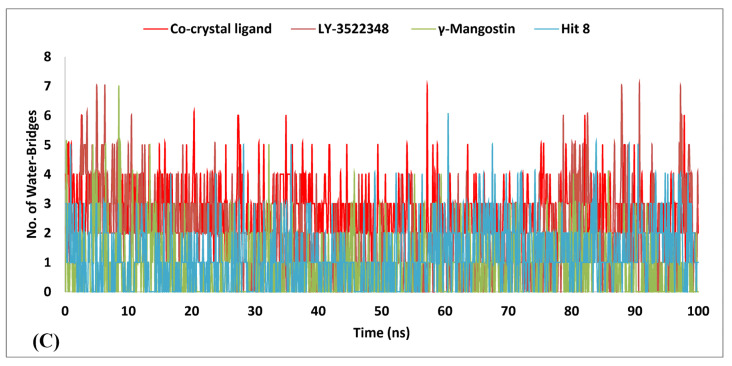
Analysis of H-bonds (**A**), hydrophobic contacts (**B**), and water bridges (**C**) for the co-crystal ligand, LY-3522348, γ-Mangostin, and hit **8** during 100 ns MD simulations.

To gain a deeper understanding of the compounds’ stability, we performed an individual RMSF analysis for the binding site residues. Key residues such as Gly106, Asn107, and Arg108 exhibited varying degrees of flexibility across different systems, as reflected in their RMSF values. The co-crystal ligand showed the highest flexibility, with values ranging from 2.7 Å to 3.8 Å, indicating significant dynamic motion at the binding pocket. The apoprotein displayed moderate fluctuations, with RMSF values between 1.7 Å and 2.2 Å. γ-Mangostin exhibited a slightly lower flexibility compared to the apoprotein, with RMSF values ranging from 1.9 Å to 2.3 Å. In contrast, hit **8** demonstrated the lowest flexibility among all systems, with values between 1.2 Å and 1.5 Å, highlighting its strong and stable interaction at these critical residues. Hit **7** showed a slightly higher flexibility than hit **8**, with values ranging from 2.2 Å to 2.8 Å, while LY-3522348 maintained a moderate flexibility, with RMSF values ranging from 1.7 Å to 2.3 Å. These variations underscore the superior stability of hit **8** and, to a lesser extent, hit **7**, compared to γ-Mangostin, LY-3522348, and the co-crystal ligand. Lys193 exhibited varying levels of flexibility across the analyzed systems, with hit **8** showing the lowest RMSF value of 1.35 Å, indicating a superior stability. In comparison, the co-crystal ligand exhibited the highest flexibility at 2.1 Å, followed by the apoprotein at 1.8 Å, γ-Mangostin at 1.84 Å, and LY-3522348 at 1.4 Å, reflecting moderate to low fluctuations. Hit **8** demonstrated the best stability at residues Gly255 and Gly257, with RMSF values of 0.94 Å and 0.78 Å, respectively, outperforming all other compounds. This highlighted its strong interaction and minimal flexibility at these critical binding site residues. Additionally, the RMSF value of 0.76 for Phe260, which forms part of the hydrophobic subpocket [21], reflected low structural fluctuations, indicating stable binding in this region during the MD simulation. The interaction with Phe260 contributed to the stability of the hydrophobic subpocket, enhancing the overall binding of hit **8**. Regarding Asp27 (a critical residue in subunit B) [23], hit **8** demonstrated the best stability, with an RMSF value of 1.4 Å, outperforming all other compounds, including the co-crystal structure (3.0 Å), γ-Mangostin (2.2 Å), and LY-3522348 (2.2 Å). This further underscored hit **8**′s superior interaction and stability within the binding site. Moreover, both Cys282 and Phe245, critical residues in the enzyme’s active site involved in the formation of the water bridge interactions seen in all currently developed inhibitors [5,21,23,24], showed RMSF values of 1.1 Å and 1.8 Å, respectively, when they interacted with hit **8**. Compared to the other compounds, these values were lower, indicating minimal structural fluctuations at these key residues and highlighting their stability. Similarly, Thr253, another critical residue in the enzyme’s active site, showed an RMSF value of 1.2 Å when interacting with hit **8**, lower than that observed for other compounds. This indicated minimal structural fluctuations at Thr253, further suggesting an enhanced stability at this key residue. To conclude, the RMSF analysis clearly demonstrated that hit **8** exhibited a superior stability at critical binding site residues, including Lys193, Gly106, Asn107, Arg108, Gly255, Gly257, Phe260, Asp27, Cys282, Phe245, and Thr253, compared to other compounds, highlighting its potential as a highly stable and effective inhibitor. Its minimal structural fluctuations at these key residues suggested that hit **8** significantly enhanced the enzyme–inhibitor interaction, offering a promising therapeutic advantage in targeting the enzyme’s active site. Given its lower RMSD average of 2.7 Å and its exceptional ability to stabilize binding site residues compared to the reference ligands, hit **8** was selected for a comprehensive interaction analysis, as presented in Table 3 and Figure 13. This analysis provides detailed insights into the factors contributing to its superior performance over the other hits, thereby reinforcing its potential as a promising lead compound. Hit **8** exhibited an average hydrogen bond contact of 0.5, as shown in Figure 13A, which was similar to the co-crystal ligand, but significantly lower than γ-Mangostin (2.7) and LY-3522348 (1.1), indicating a comparatively weaker hydrogen bond formation. However, hit **8** compensated for this with exceptional hydrophobic interactions, as seen in Figure 13B, achieving the highest average (2.0) and maximum (6.0) values among all hits, demonstrating its strong ability to stabilize the binding site through non-polar interactions. In terms of water bridge contacts, depicted in Figure 13C, hit **8** recorded an average of 1.2, outperforming LY-3522348 (0.8) but falling behind γ-Mangostin (1.8) and the co-crystal ligand (2.7). This suggested moderate water-mediated interactions. Despite its lower hydrogen bond and water bridge averages, the superior hydrophobic contacts of hit **8** made it a strong candidate, reflecting a balanced interaction profile and promising potential for effectively stabilizing the KHK-C binding site. Hit **8**, as shown in Supplementary Figure S2A, exhibited persistent hydrophobic interactions, which were dominated by several hydrophobic residues in the binding site, including Ala224, Ala226, Ala230, Ala244, Pro246, Pro247, Val250, Ala256, Phe260, Ala285, and Cys289. These interactions were not observed in the reference ligands in Supplementary Figure S2B–D, highlighting the unique binding profile of hit **8**. The hydrophobic interactions contributed significantly to its ability to stabilize the binding site through non-polar contacts, complementing the interaction profiles depicted in Figure 13A–C. Despite having a lower average for hydrogen bonds and water bridge contacts, the superior hydrophobic interactions of hit **8** positioned it as a strong candidate for stabilizing the KHK-C binding site effectively.

### 2.6. Quantum Mechanical Calculations

**Figure 14:**
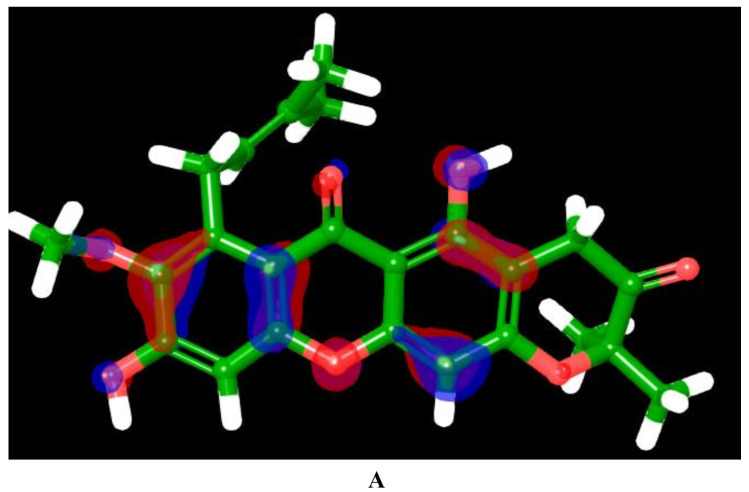

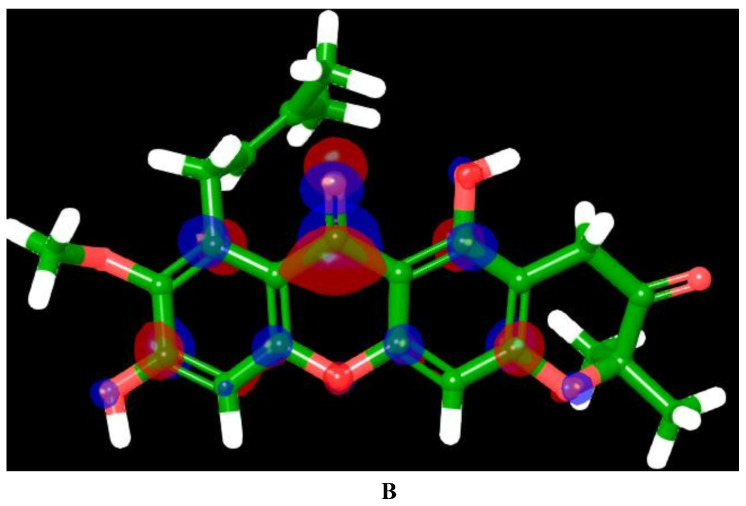
Spatial Distribution of HOMO (**A**) and LUMO (**B**) orbitals in hit **8**.

The molecular orbital and electronic properties of investigational hits are typically analyzed using quantum mechanical approaches [64,65]. In the present study, hit **8** emerged as the top-performing compound among the identified hits, demonstrating the best binding affinity, enhanced pharmacokinetics, favorable physicochemical properties, a high absorption rate, and an excellent metabolic stability. Additionally, its stable dynamic behavior within the KHK-C binding site further solidified its potential. As a result, it was subjected to quantum mechanical calculations to investigate its electronic properties, interaction energies, and reactivity, aiming to gain deeper insights into its binding mechanism and optimize its drug-like characteristics. A molecule’s electron affinity is directly related to its LUMO energy, while its ionization potential is associated with its HOMO [66]. The HOMO energy of hit **8** was −6.02 eV, and the LUMO energy was −1.62 eV, resulting in a HOMO–LUMO Gap (HLG) of 4.4 eV (Table 4). This gap indicated a moderate electronic stability and reactivity, highlighting the molecule’s ability to participate in electron transfer processes [67]. Its solvation energy of −17.80 eV reflected strong interactions with the solvent, signifying a significant stabilization effect in the solvated state. This property suggests potential applications in environments where solvation effects are critical, such as in biological or catalytic systems. Furthermore, other quantum mechanical descriptors were calculated following equations published in the literature [68]. The chemical hardness (η) (2.2 eV) and softness (σ) (0.45 eV^−1^) (Table 4) underscore the molecule’s relatively rigid electronic structure, while the global electrophilicity index (ω) (3.32 eV) demonstrates its capacity as an effective electron acceptor in chemical reactions. The localization of the molecular orbitals (Figure 14) provides further insight into the electronic characteristics of hit **8**. The HOMO energy was predominantly found on the hydroxyl (OH) groups, the oxygen atom of the methoxy group, the oxygen atom of the xanthene ring, the carbonyl group of the xanthone ring, and the aromatic rings of the xanthene framework. These regions represent the molecule’s electron-donating centers, contributing to its reactivity and stability under oxidative conditions. In contrast, the LUMO energy was primarily localized on the oxygen atom of the pyrano ring, the carbonyl group of the xanthone ring, the oxygen atom of the xanthene ring, and the two hydroxyl groups. These regions act as electron-accepting centers, facilitating interactions with nucleophiles and determining the molecule’s reactivity in reduction processes. The electronic properties of hit **8** are directly influenced by its structural features. The xanthene backbone, with its conjugated π-system, plays a pivotal role in defining the HOMO–LUMO gap, allowing for extensive electron delocalization and contributing to the compound’s moderate stability. The presence of electron-donating groups, such as the 8-methoxy and 5,9-dihydroxy substituents, lowers the HOMO energy, enhancing oxidative stability while preserving reactivity. The prenyl side chain and dimethyl groups introduce steric effects, shielding the molecule’s reactive centers and further stabilizing the structure. Collectively, these features contribute to the molecule’s high electrophilicity, moderate chemical hardness, and predictable reactivity profile. The significant solvation energy of −17.80 kcal/mol is consistent with the structural and electronic features of hit **8**. The conjugated xanthene scaffold and its polar functional groups enhance the molecule’s polarizability, enabling strong solvent interactions and stabilizing the solvation shell. The hydroxyl and methoxy substituents act as hydrogen bond donors and acceptors, amplifying the solvation stability by forming dipolar and hydrogen bonding interactions with the solvent. The hydrophobic prenyl chain balances the molecule’s solvation dynamics by reducing excessive solubility. This synergy between the electronic and structural features explains the molecule’s stable behavior in solution, highlighting its potential applications in various chemical and biological systems.

## 3. Material and Methods

In this study, several computational tools from the Schrödinger suite (Schrödinger, LLC, Version Number: Schrödinger Software Suite 2023-1, New York, NY 10036, USA) were utilized, including the Protein Preparation Wizard [69], LigPrep [70], Glide [71,72], Prime [73,74], Qikprop [75], Jaguar [76], and Desmond [77]. These tools were accessed utilizing the Maestro graphical interface [78].

### 3.1. Target Selection and Validation

A literature search identified multiple high-resolution crystal structures from the Protein Data Bank (https://www.rcsb.org/) accessed on 20 November 2024 for use in docking studies, with **8UG1** (1.99 Å), 8OMJ (1.98 Å), and **9FHD** (1.85 Å) being shortlisted for their superior resolution. However, **9FHD** was not available for download at the time of analysis. Consequently, the **8UG1** and **8OMJ** structures were retained for comparative evaluation. To assess the reliability of the selected structures, several criteria were considered. A protein reliability analysis was performed based on key structural quality metrics, including R-values, binding site packing, and steric clash evaluation. The R-values (R-Free and R-Work) were used as indicators of crystallographic quality and model accuracy.

### 3.2. Molecular Docking

Virtual screening of prenylated xanthones, consisting of 1383 molecules collected from the COlleCtion of Open NatUral producTs (COCONUT) Database (https://coconut.naturalproducts.net/) accessed on 25 November 2024, PubChem Database (https://pubchem.ncbi.nlm.nih.gov/) accessed on 25 November 2024, and the published literature [36,40,41,42,43], was performed using the Virtual Screening Workflow (VSW) from the Schrödinger suite. The compound library was retrieved using a Fingerprint Tanimoto-based 2D similarity search, adjusting the threshold to a 95% similarity, with the text query α-Mangostin. The library was then converted into 3D formats, followed by optimization using the OPLS4 force field. The LigPrep module (Schrödinger Release 2023-1) was employed to maintain the ligands’ original chirality and prepare the structures for docking. The ionization states of the ligands were generated using Epik at pH 7.00 ± 2 units, and for each ligand, one low-energy conformer was generated. The 3D crystal structure of human KHK-C complexed with the reference ligand (PDB code: **8UG1**) was retrieved from the Protein Data Bank (www.rcsb.org) accessed on 25 November 2024. The Protein Preparation Wizard (PrepWizard) (Schrödinger Release 2023-1) was used to refine and optimize the protein structure for further analysis. After removing water molecules greater than 5 Å, the co-crystal ligand in chain A was retained at the enzyme’s catalytic site, while the ligand in chain B was removed. Optimization and energy minimization were performed using the OPLS4 force field. A receptor grid box was generated around the coordinates of the reference ligand in the binding site of chain A using the receptor grid generation tool in Maestro. The prepared ligands were subsequently evaluated against the optimized target protein using the multi-step receptor docking process in the Glide module of Schrödinger (Schrödinger Release 2023-1). The screening began with Glide’s high-throughput virtual screening (HTVS) mode to filter the compound library, followed by a more refined screening using the standard precision (SP) mode. For the final step, the most accurate docking predictions were generated using extra precision (XP) mode. Each ligand was assigned its best pose, and their ranking was determined based on the Glide docking score. The clinical candidate LY-3522348, γ-Mangostin, and α-Mangostin were docked for comparative studies. Additionally, the co-crystal ligand was re-docked several times, and the RMSD values were calculated to assess the accuracy of the docking procedure.

### 3.3. Binding Free Energy Calculations

The Prime module (Schrödinger Release 2023-1), integrated with Schrödinger, was used to calculate the binding free energy of the receptors and docked ligands. The Pose Viewer Files (PVFs) generated after docking served as inputs to compute the binding free energy for each compound. The VSGB 2.0 solvation model and OPLS4 force field were applied to derive the free energy descriptors. The default settings for Prime were used. The MM–GBSA ∆G binding energy score was then employed to rank the ligands based on their binding affinity. MM–GBSA calculations were performed for the co-crystal ligand, clinical candidate LY-3522348, γ-Mangostin, and α-Mangostin to evaluate their binding free energies. The energy terms contributing to the binding affinities for the top hits were analyzed to gain insights into the molecular interactions and identify the key factors influencing the binding process.

### 3.4. ADMET Profiling

In this study, QikProp (Schrödinger Release 2023-1) was used to assess the ADMET profiles and drug-likeness descriptors of the top hits with high binding free energy scores, along with the reference drug candidate LY-3522348 and the original ligand γ-Mangostin. Ligand structures were prepared using the LigPrep tool to optimize their 3D geometries and assign protonation states at a physiological pH of 7.4. Predictions were made using the default settings of QikProp, which include evaluations of key parameters such as LogP, solubility (LogS), CNS permeability, human oral absorption, and potential toxicological risks. All calculations were performed in standalone mode within the Schrödinger software (Schrödinger Release 2023-1) environment. The program provides recommended ranges for various properties and descriptors of small molecules, based on an analysis of 95% of known drugs. The results were exported into an MS Excel file, which included the principal descriptors and ADME predictions.

### 3.5. Molecular Dynamics Simulations

Molecular dynamics (MD) simulations were conducted using the Desmond MD simulation package to study the best ligand–target complexes, including the co-crystal ligand, the clinical candidate LY-3522348, γ-Mangostin, and top-performing hits from the docking studies (hits 7, 8, 9, 13, and 15). These hits demonstrated a higher binding affinity than LY-3522348, along with favorable absorption rates and metabolic stability. The SPC solvation model was applied, and each system was placed in an orthorhombic water box with dimensions of 10 Å × 10 Å × 10 Å to ensure full solvent coverage around the complexes. Na^+^ counter ions were added to neutralize the net charges, and 0.15 M NaCl was included to maintain system neutrality following a method from the literature [79]. The systems were then energy-minimized and pre-equilibrated using the default relaxation protocol in the Desmond package. Then, they were minimized and pre-equilibrated again before the simulation was run using the default relaxation protocol in the Desmond package. The temperature of the system was maintained at 300 K and the atmospheric pressure at 1 bar using an isothermal–isobaric (NPT) ensemble [80]. Overall, a simulation of 100 ns was carried out, and 1000 frames of data were generated every 100 ps as recording intervals. The simulation event was analyzed through the Simulation Interaction Diagram (SID) available at the Schrödinger package (Schrödinger Release 2023-1). From the trajectory output, root-mean-square fluctuation (RMSF), RMSD, and protein–ligand contacts (P–L contact) were also analyzed.

### 3.6. Quantum Mechanical Calculations

Quantum chemical calculations for hit **8** were conducted using the Density Functional Theory (DFT) method available in the Jaguar module of the Schrödinger suite (Schrödinger Release 2023-1). These calculations focused on evaluating electronic molecular properties, including electron density and energy levels of the HOMO and LUMO orbitals [81]. The calculated energy values were further used to determine several quantum chemical descriptors, such as the energy gap (HLG), chemical hardness, chemical softness, electronegativity, and global electrophilicity index, in accordance with established equations reported in the literature [68]. DFT calculations were carried out using a combination of traditional functionals, as follows: Becke’s three-parameter functional and the Lee–Yang–Parr functional (B3LYP), supplemented with a dispersion correction term (D3), collectively referred to as B3LYP–D3 [81]. Electron-deficient surfaces are marked by a blue color, whereas electron-rich ones are indicated by a red color.

## 4. Conclusions

In this study, we identified several novel analogs with potent inhibitory effects on the KHK-C enzyme, surpassing α-Mangostin in binding affinity. Hit **8**, in particular, demonstrated the highest binding free energy and exhibited favorable pharmacokinetic properties, including a 100% predicted oral absorption and an enhanced metabolic stability. Molecular dynamics simulations confirmed the stability of hit **8** in the KHK-C binding site, reinforcing its potential as a promising therapeutic candidate. Quantum mechanical calculations further highlighted its broad applications. These findings provide a strong basis for further experimental validation and the development of KHK-C inhibitors for the treatment of fructose-driven metabolic disorders.

## Figures and Tables

**Table 1:** Docking scores, molecular interactions, and bond lengths for the top 16 hits, co-crystallized ligand, clinical candidate LY-3522348, γ-Mangostin, and α-Mangostin.

NO	PubChem ID	Docking Score (kcal/mol)	Binding Energy (kcal/mol)	Interactions	
				H-Bond	Water Bridge
Co-crystal ligand		−9.371	−63.37	Glu227 (2.21 Å)	Ala226 (2.15 and 2.19 Å) Phe245 (2.05 and 1.91 Å) Cys282 (2.05 and 1.78 Å)
LY-3522348		−8.409	−45.36	Glu227 (1.91 Å) Thr253 (2.16 Å)	Phe245 (1.88 and 1.91 Å) Cys282 (1.88 and 1.78 Å)
γ-Mangostin		−7.644	−38.58	Ala226 (1.80 Å)	Asp27(1.63 and 2.01 Å) Phe245 (2.57 and 1.91 Å) Cys282 (2.57 and 1.78 Å)
α-Mangostin		−5.992	−37.34	Glu173 (1.95 Å)	Lys193 (2.17 and 2.43 Å) Ala226 (2.06 and 2.19 Å)
**1**	163184041	−10.791	−49.45	Ala226 (1.89 Å)	Asp27 (2.05 and 2.02 Å) Asp27 (1.57 and 2.02 Å) Phe245 (2.28 and 1.91 Å) Cys282 (2.28 and 1.78 Å)
**2**	–	−10.447	−40.44	Asn107 (2.31 Å) Glu227 (2.22 Å) Glu227 (2.15 Å) Glu29 (2.42 Å) Glu29 (2.17 Å) Asp194 (2.56 Å) Asp194 (2.69 Å)	Lys193 (1.98 and 2.43 Å)
**3**	129847812	−9.904	−54.11	Glu173 (2.11 Å) Arg108 (2.47 Å)	Phe245 (2.07 and 1.91 Å) Cys282 (2.07 and 1.78 Å) Asp27 (2.49 and 2.02 Å) Lys193 (2.27 and 2.43 Å)
**4**	11302345	−9.767	−37.36	Ala226 (1.88 Å)	Asp27 (1.75 and 2.02 Å) Ala226 (1.63 and 2.19 Å) Phe245 (2.31 and 1.91 Å) Cys282 (2.31 and 1.78 Å)
**5**	11530321	−9.626	−49.86	–	Asp27 (2.33 and 2.02 Å) Phe245 (1.97 and 1.91 Å) Cys282 (1.97 and 1.78 Å)
**6**	11772726	−9.444	−44.47	Asp27 (2.16 Å)	Lys193 (2.29 and 2.43 Å) Glu227 (1.83 and 2.01 Å) Phe245 (2.19 and 1.91 Å) Cys282 (2.19 and 1.78 Å)
**7**	129844441	−9.411	−55.13	Glu173 (1.94 Å)	Asp27 (2.32 and 2.02 Å) Lys193 (2.14 and 2.43 Å) Ala226 (1.96 and 2.19 Å) Phe245 (1.99 and 1.91 Å) Cys282 (1.99 and 1.78 Å)
**8**	5495931	−9.380	−61.30	Asp194 (2.48 Å)	Lys193 (1.77 and 2.43 Å) Phe245 (2.22 and 1.91 Å) Cys282 (2.22 and 1.78 Å)
**9**	5464633	−8.999	−53.01	–	Ala226 (1.83 and 2.19 Å) Phe245 (1.73 and 1.91 Å) Cys282 (1.73 and 1.78 Å)
**10**	15293189	−8.992	−45.08	Glu173 (2.19 Å) Gly255 (2.26 Å)	Ala226 (2.78 and 2.19 Å)
**11**	5281633	−8.991	−45.02	–	Asp27 (2.31 and 2.02 Å) Ala226 (2.28 and 2.19 Å) Ala226 (2.08 and 2.19 Å) Phe245 (1.95 and 1.91 Å) Cys282 (1.95 and 1.78 Å)
**12**	10092134	−8.978	−43.11	–	Asp27 (1.72 and 2.02 Å) Ala226 (2.32 and 2.19 Å)
**13**	10001484	−8.233	−59.13	–	Lys193 (2.65 and 2.43 Å) Phe245 (2.12 and 1.91 Å) Cys282 (2.12 and 1.78 Å)
**14**	10245099	−8.231	−44.84	Asp27 (2.09 Å)	Asp27 (1.92 and 2.02 Å) Phe245 (1.77 and 1.91 Å) Cys282 (1.77 and 1.78 Å)
**15**	162856452	−8.093	−45.51	Thr253 (2.76 Å)	Ala226 (1.85 and 2.19 Å) Phe245 (1.94 and 1.91 Å) Cys282 (1.94 and 1.78 Å)
**16**	132988553	−8.012	−42.75	–	Asp27 (1.80 and 2.02 Å) Ala226 (2.05 and 2.19 Å) Ala226 (2.33 and 2.19 Å)

**Table 2:** Docking scores and binding energies of the top 5 marine-derived natural products.

Compounds	Docking Score (kcal/mol)	Binding Free Energy (kcal/mol)
**1**	−9.395	−22.74
**2**	−7.402	−32.06
**3**	−8.810	−44.39
**4**	−7.704	−37.87
**5**	−8.894	−51.83

**Table 3:** RMSD, RMSF, and ligand–protein Contacts (H-bonds, water bridges, hydrophobic, and Pi–cation interactions) for Apo form, co-crystal ligand, LY-3522348, γ-Mangostin, and top 5 hits over a 100 ns MD simulation.

KHK-C Complex	Apo	Co-Crystal Ligand	LY-3522348	γ-Mangostin	Hit 7	Hit 8	Hit 9	Hit 13	Hit 15
PL-RMSD (Å)									
Average	2.9	4.4	2.9	2.9	2.6	2.7	3.6	3.4	4.2
Maximum	4.8	6.8	4.6	4.8	4.2	4.9	5.1	4.9	6.3
Minimum	1.3	1.3	1.3	1.2	1.1	1.3	1.3	1.6	1.3
P-RMSF (Å)									
Average	1.6	2.1	1.6	1.5	1.6	1.4	1.5	1.6	1.7
Maximum	5.5	13.7	14.3	9.5	8.9	11.2	12.3	8.0	7.8
Minimum	0.6	0.7	0.6	0.6	0.5	0.5	0.6	0.6	0.5
H-bond contacts									
Average	-	0.5	1.1	2.7	-	0.5	-	-	-
Maximum	-	3.0	3.0	4.0	-	3.0	-	-	-
Minimum	-	0.0	0.0	0.0	-	0.0	-	-	-
Hydrophobic contacts									
Average	-	0.5	0.8	1.2	-	2.0	-	-	-
Maximum	-	3.0	4.0	5.0	-	6.0	-	-	-
Minimum	-	0.0	0.0	0.0	-	0.0	-	-	-
Water bridge contacts									
Average	-	2.7	1.8	0.8	-	1.2	-	-	-
Maximum	-	7.0	7.0	7.0	-	6.0	-	-	-
Minimum	-	0.0	0.0	0.0	-	0.0	-	-	-

**Table 4:** Quantum mechanical descriptors of hit 8: electronic and solvation properties.

Property	Solvation Energy kcal/mol	HOMO (eV)	LUMO (eV)	HLG	Electron Affinity (eV)	Ionization Potential (eV)	Chemical Hardness (eV)	Chemical Softness (eV^−1^)	Electronegativity (eV)	Global Electrophilicity Index (eV)
**Hit 8**	−17.80	−6.02	−1.62	4.4	1.62	6.02	2.2	0.45	−3.82	3.32

## Data Availability

The original contributions presented in this study are included in the article/Supplementary Materials. Further inquiries can be directed to the corresponding author.

## Supplementary Materials

The following supporting information can be downloaded at: https://www.mdpi.com/article/10.3390/ph18010126/s1, Figure S1: 2D interaction map of ligand binding; Figure S2: Histogram of interactions for Hit **8** (**A**), γ-Mangostin (**B**), LY-3522348 (**C**), and the co-crystal ligand (**D**); Table S1: QikProp-generated physicochemical and pharmacokinetic properties of the top 16 hits. The figure compares the interaction profiles of the compounds, highlighting the distinctive hydrogen bond, hydrophobic, and water-bridge contacts.
