# Supplementary material for: Examining Prenylated Xanthones as Potential Inhibitors Against Ketohexokinase C Isoform for the Treatment of Fructose-Driven Metabolic Disorders: An Integrated Computational Approach
Source: Pharmaceuticals (Basel). 2025 Jan 18;18(1):126. doi: 10.3390/ph18010126 (PMC11768319; doi:10.3390/ph18010126)
Supplement: Supplementary file 1 [file pharmaceuticals-18-00126-s001.zip › pharmaceuticals-3437767-supplementary.pdf]

Supplementary Material

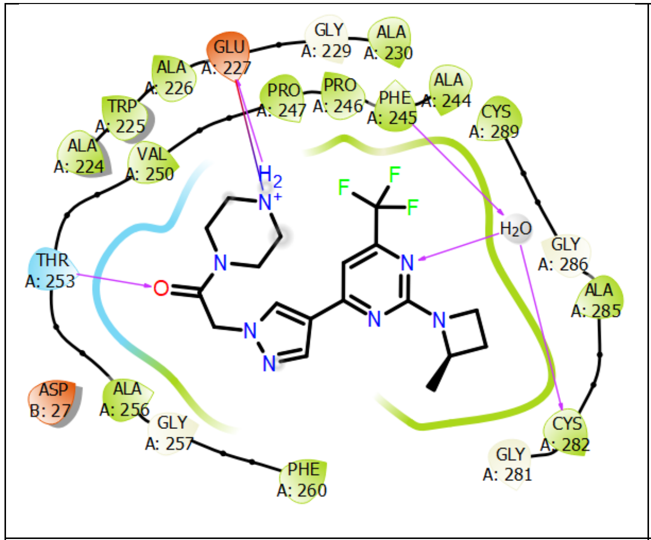

LY-3522348

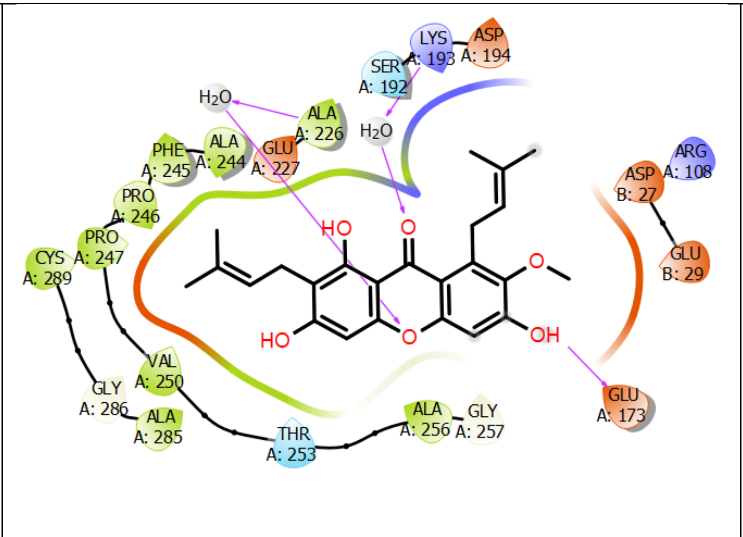

Alpha-Mangostin

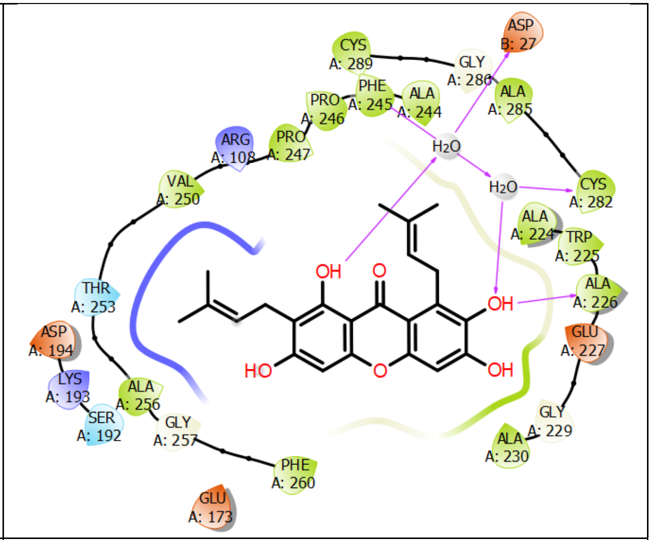

Gamma-Mangostin

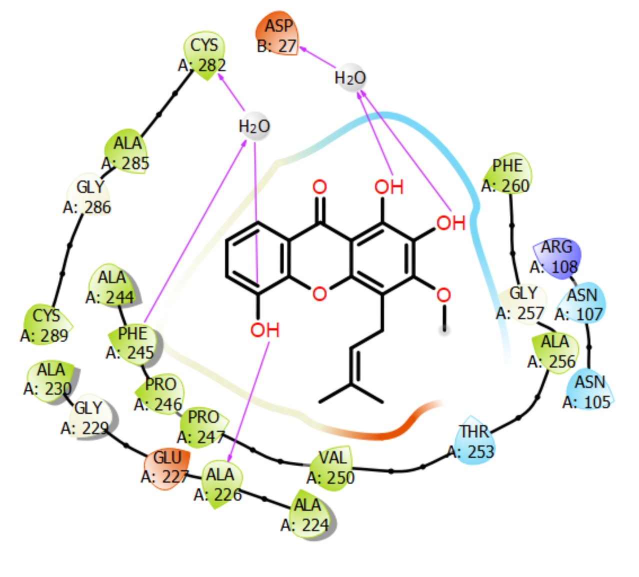

Hit1

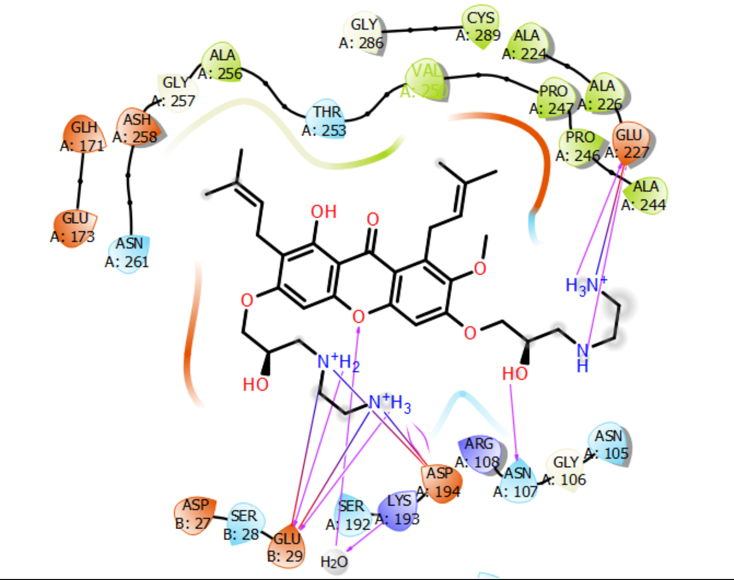

Hit 2

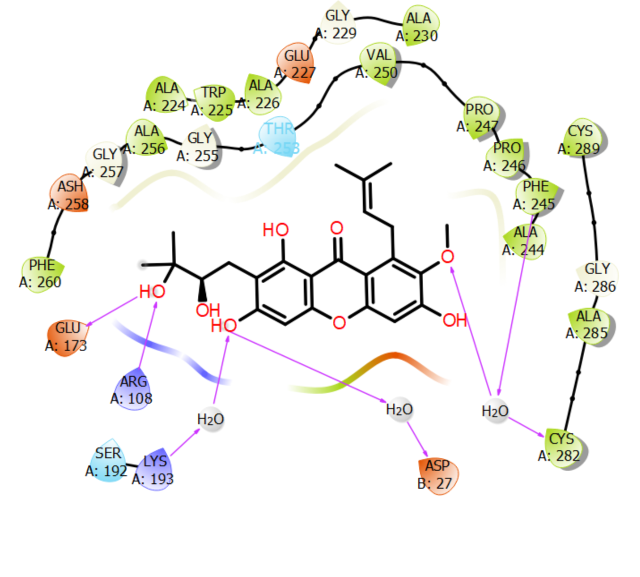

Hit 3

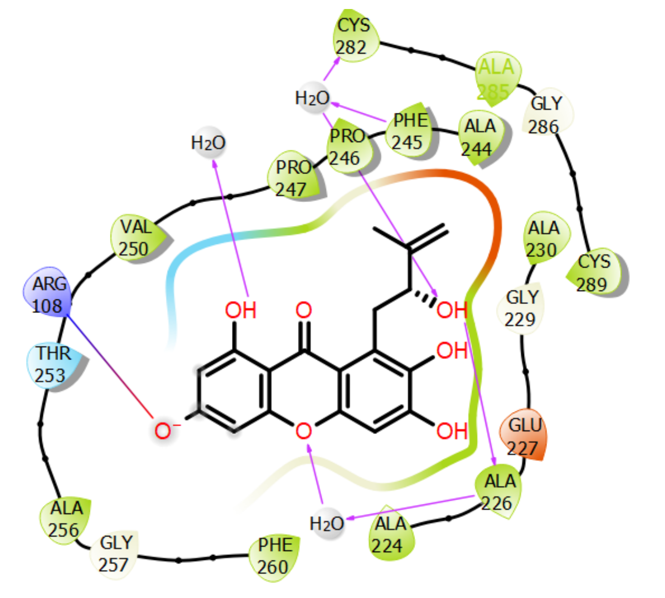

Hit 4

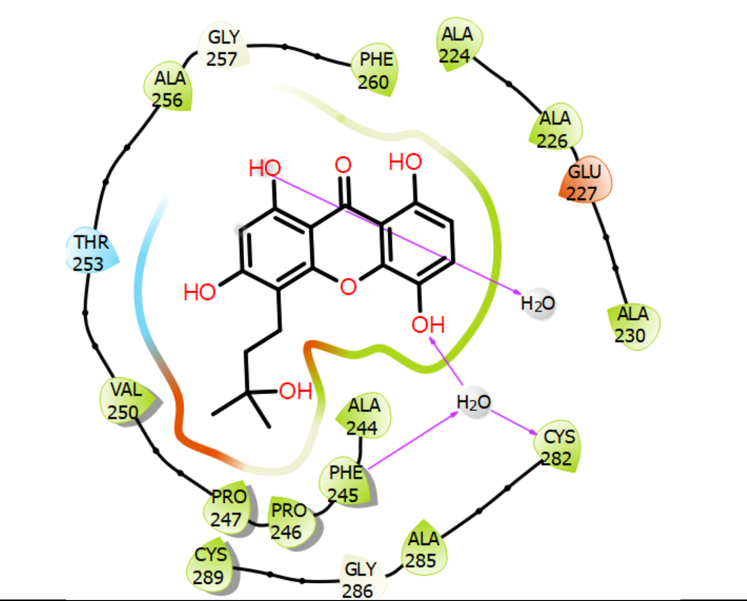

Hit 5

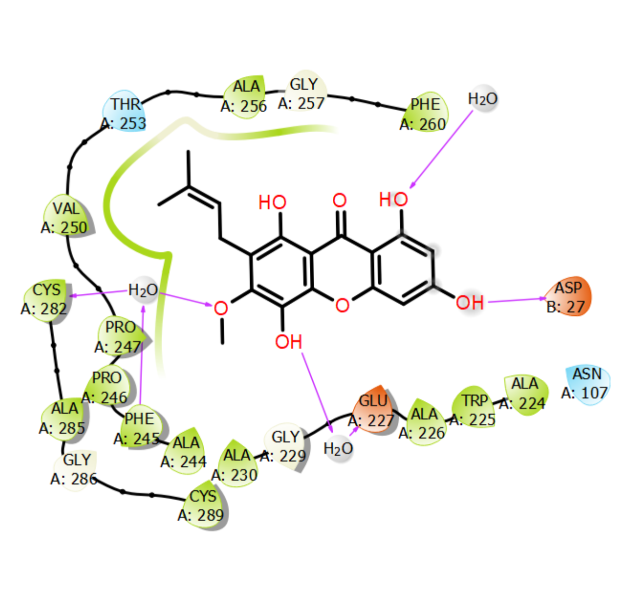

Hit 6

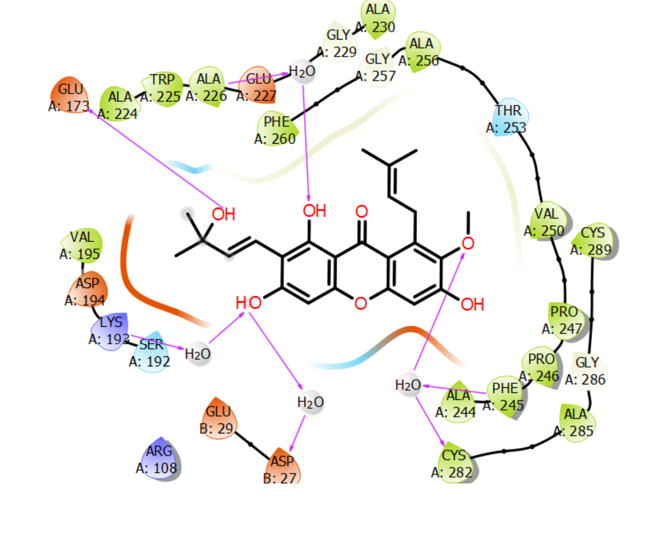

Hit 7

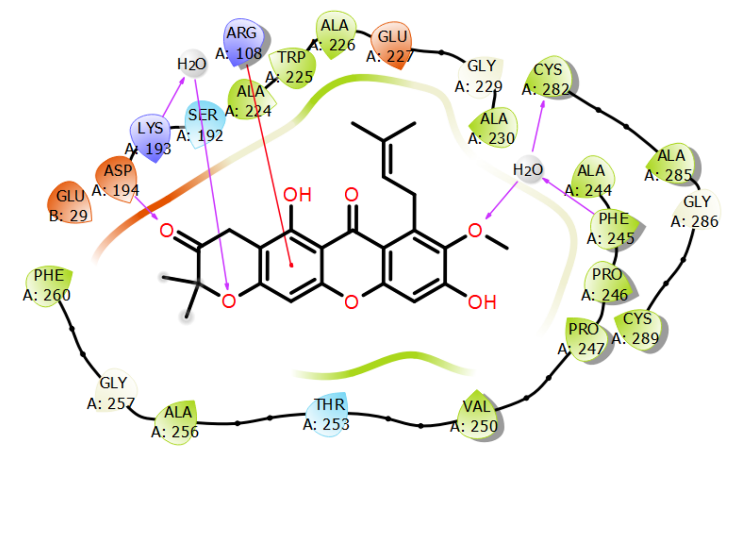

Hit 8

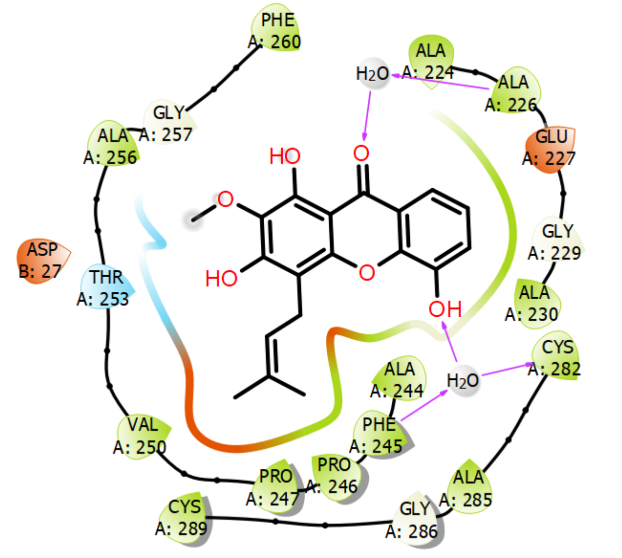

Hit 9

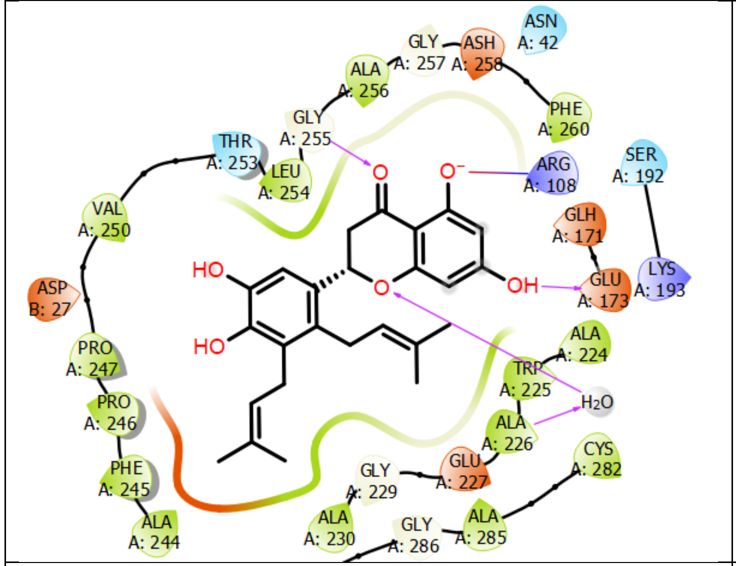

Hit 10

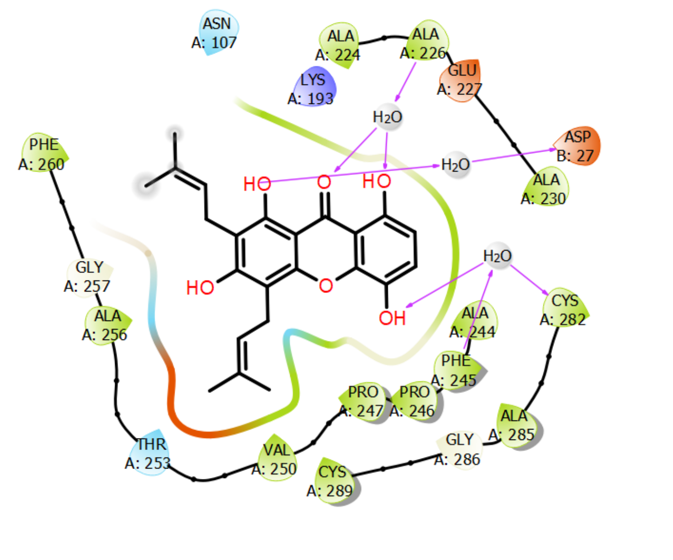

Hit 11

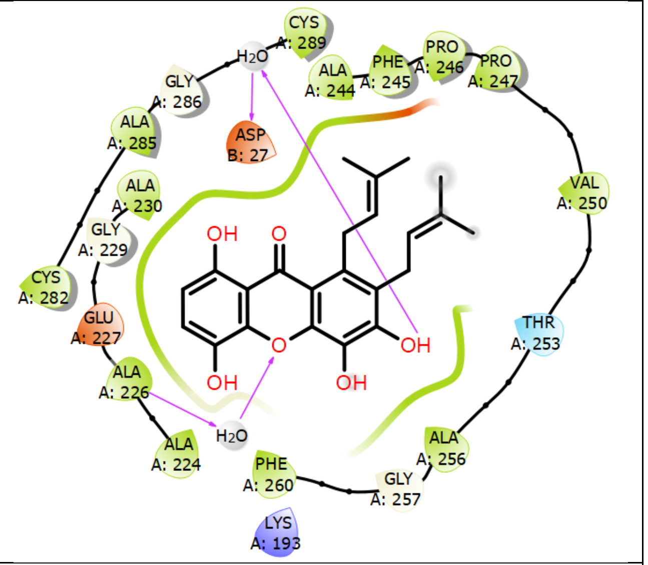

Hit 12

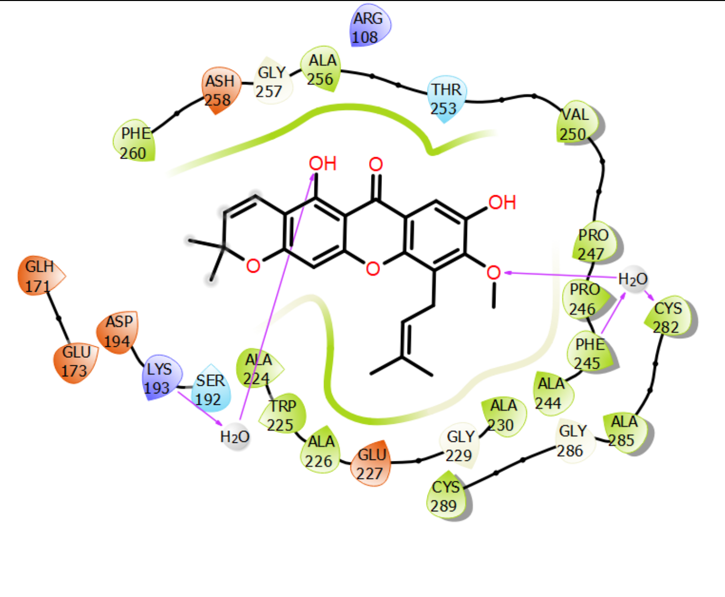

Hit 13

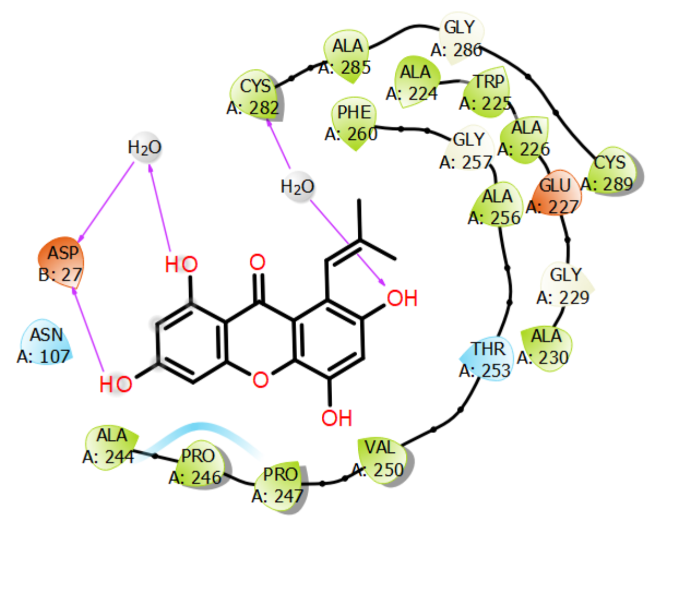

Hit 14

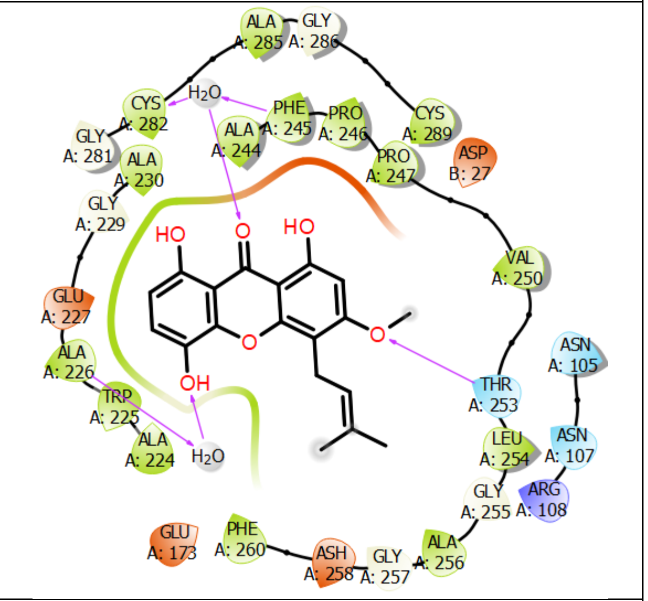

Hit 15

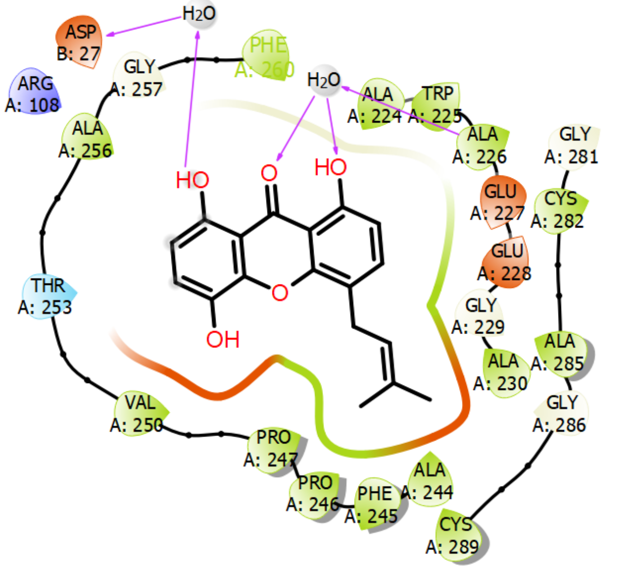		
**Hit 16**		

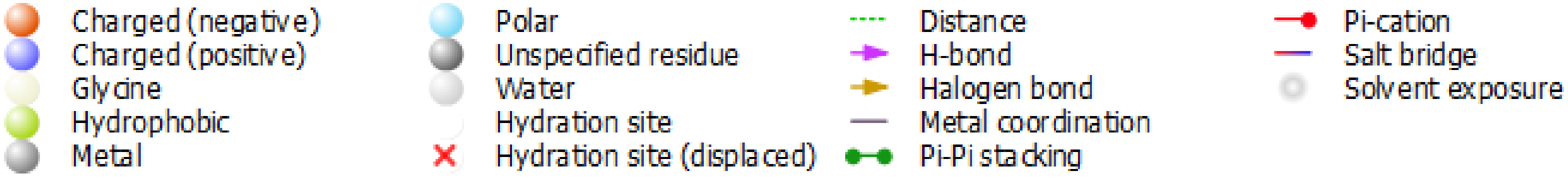

Supplementary Figure S1. 2D interaction map of ligand binding.

Table S1: QikProp-generated physicochemical and pharmacokinetic properties of the top 16 hits.

Hit	#stars	CNS	mol_MW	SASA	FOSA	FISA	PISA	donorHB	accptHB	QPlogPo/w	QPlogS	CIQPlogS	QPlogHERG	QPPCaco	QPlogBB	QPPMDCK	#metab	QPlogKhsa	%OralAbsorption	PSA	RuleOfFive	RuleOfThree
**LY-3522348**	0	1	409.4	700.5	390.5	103.4	92.32	1	8	2.33	-4.19	-3.207	-4.651	165.9	0.029	534.9	1	-0.03	80.347	85.84	0	0
**γ-Mangostin**	1	-2	396.43	684.6	370.2	165.0	149.3	3	4.5	3.732	-5.466	-6.108	-5.341	269.5	-1.602	119.9	10	0.569	92.299	108.3	0	1
**1**	0.0	-2.0	342.3	573.5	241.9	159.1	172.5	2.0	4.5	2.8	-4.1	-5.1	-4.6	306.8	-1.2	138.0	7.0	0.3	87.9	101.2	0.0	1.0
**2**	8.0	-2.0	642.8	1010	654.0	265.1	90.9	8.0	12.9	1.1	-0.2	-3.1	-9.0	0.5	-3.3	0.2	18.0	-0.1	0.0	177.5	3.0	2.0
**3**	1.0	-2.0	444.5	693.3	397.2	162.8	133.4	4.0	7.0	3.0	-4.3	-6.1	-5.0	283.3	-1.7	126.6	10.0	0.2	88.6	125.8	0.0	1.0
**4**	0.0	-2.0	344.3	553.8	137.4	217.8	198.7	4.0	6.2	1.2	-2.8	-4.4	-4.7	85.2	-1.9	34.5	7.0	-0.3	68.5	127.6	0.0	1.0
**5**	0.0	-2.0	346.3	562.2	169.5	220.0	172.7	3.0	4.3	2.1	-3.4	-5.2	-4.6	81.2	-1.9	32.8	6.0	0.1	73.6	132.3	0.0	0.0
**6**	0.0	-2.0	358.3	600.5	262.4	196.3	141.8	2.0	4.3	2.8	-4.4	-5.6	-4.9	136.3	-1.7	57.4	8.0	0.3	81.4	120.0	0.0	1.0
**7**	0.0	-2.0	426.5	710.4	396.3	159.7	154.4	3.0	5.3	3.9	-5.5	-6.4	-5.4	303.2	-1.6	136.2	8.0	0.6	94.1	110.9	0.0	1.0
**8**	0.0	-1.0	424.4	683.8	427.1	123.0	133.8	1.0	6.5	3.8	-5.5	-6.2	-5.0	675.4	-0.9	323.7	7.0	0.5	100.0	106.2	0.0	1.0
**9**	0.0	-2.0	342.3	585.6	270.4	145.2	170.0	2.0	4.5	3.0	-4.3	-5.1	-4.8	415.5	-1.1	191.5	7.0	0.3	91.1	100.0	0.0	1.0
**10**	1.0	-2.0	424.5	669.3	346.6	215.3	107.4	3.0	4.8	3.6	-5.1	-6.6	-4.5	89.9	-1.9	36.6	12.0	0.7	82.8	111.4	0.0	1.0
**11**	1.0	-2.0	396.4	683.6	367.6	165.7	150.3	2.0	3.5	4.4	-6.0	-6.6	-5.3	265.8	-1.6	118.1	10.0	0.9	95.8	106.1	0.0	2.0
**12**	1.0	-2.0	396.4	673.1	335.1	193.9	144.1	3.0	4.5	3.5	-5.3	-6.1	-5.2	143.5	-1.8	60.7	10.0	0.6	85.9	112.0	0.0	1.0
**13**	0.0	-1.0	408.5	693.7	397.3	120.3	176.0	1.0	4.5	4.6	-6.4	-6.7	-5.5	715.6	-1.0	344.6	6.0	0.9	100.0	86.3	0.0	1.0
**14**	0.0	-2.0	314.3	534.3	154.2	206.6	173.5	3.0	4.5	1.6	-3.4	-4.5	-4.7	108.9	-1.6	45.0	6.0	-0.1	72.9	110.9	0.0	0.0
**15**	0.0	-2.0	342.3	597.3	275.9	164.5	156.9	1.0	3.5	3.4	-4.9	-5.6	-4.9	272.7	-1.4	121.5	7.0	0.5	90.7	99.7	0.0	1.0
**16**	0.0	-1.0	312.3	525.5	176.9	145.0	203.6	1.0	2.8	3.3	-4.0	-5.3	-4.4	417.7	-1.0	192.6	6.0	0.5	93.0	91.7	0.0	0.0

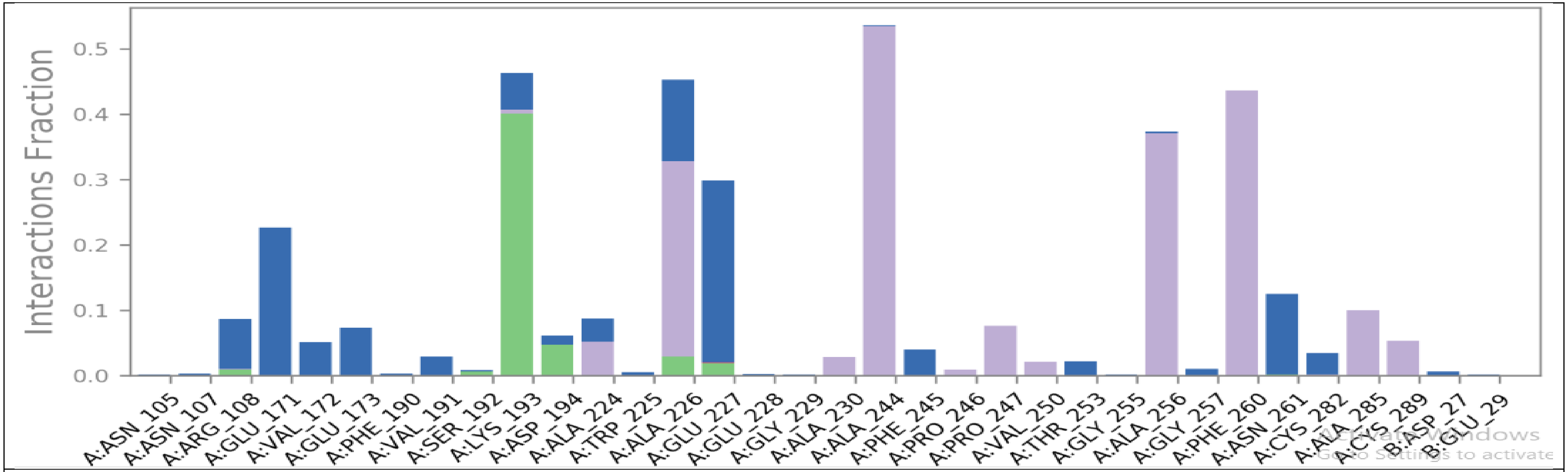

Hit 8 (A)

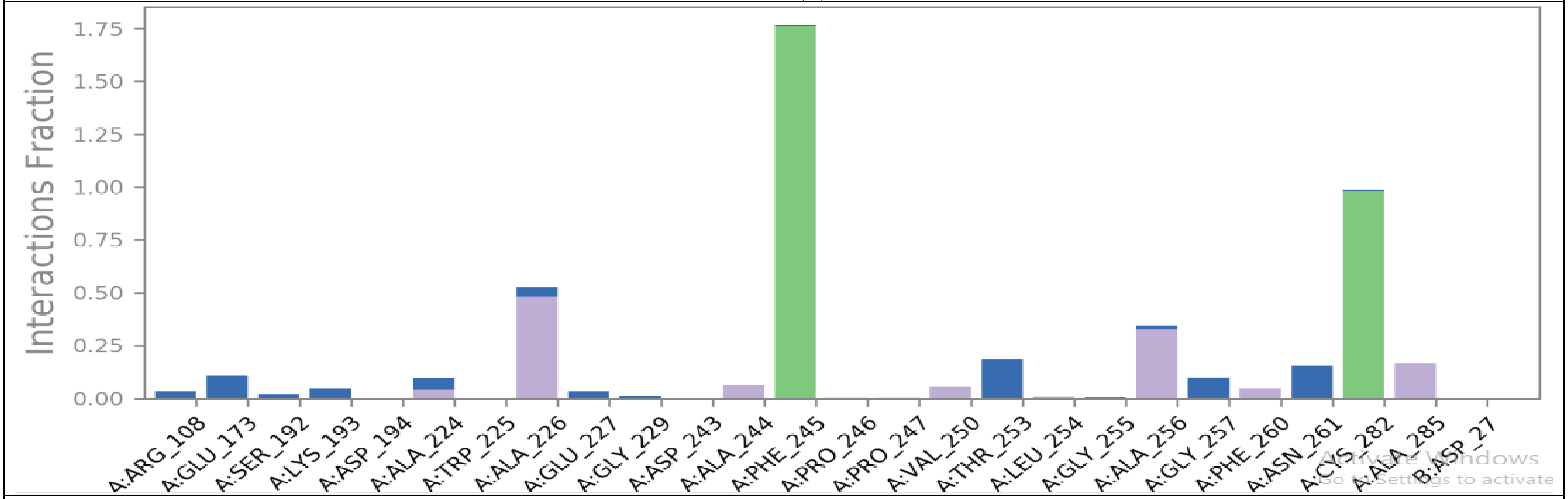

γ-Mangostin (B)

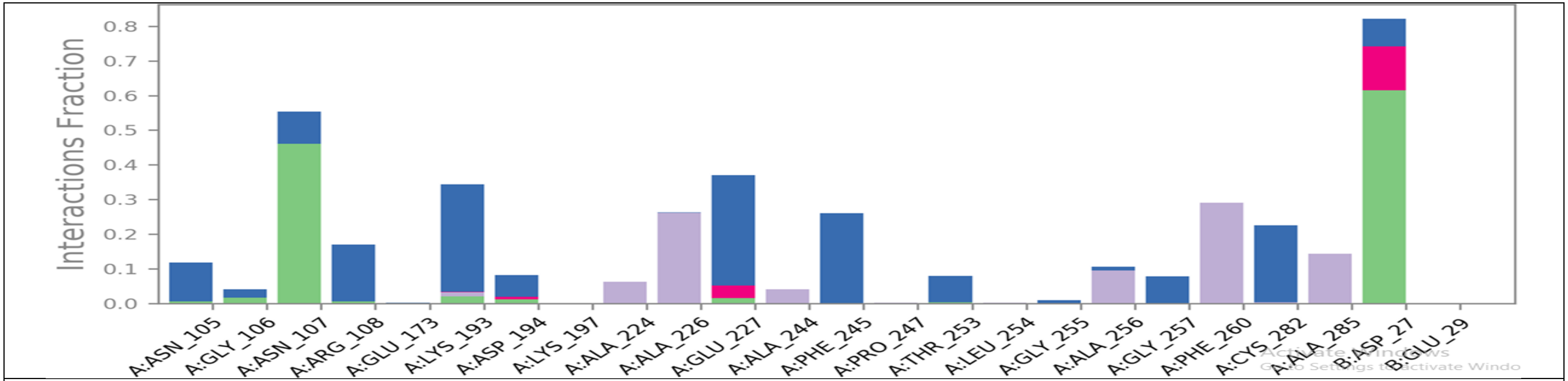

LY-3522348 (C)

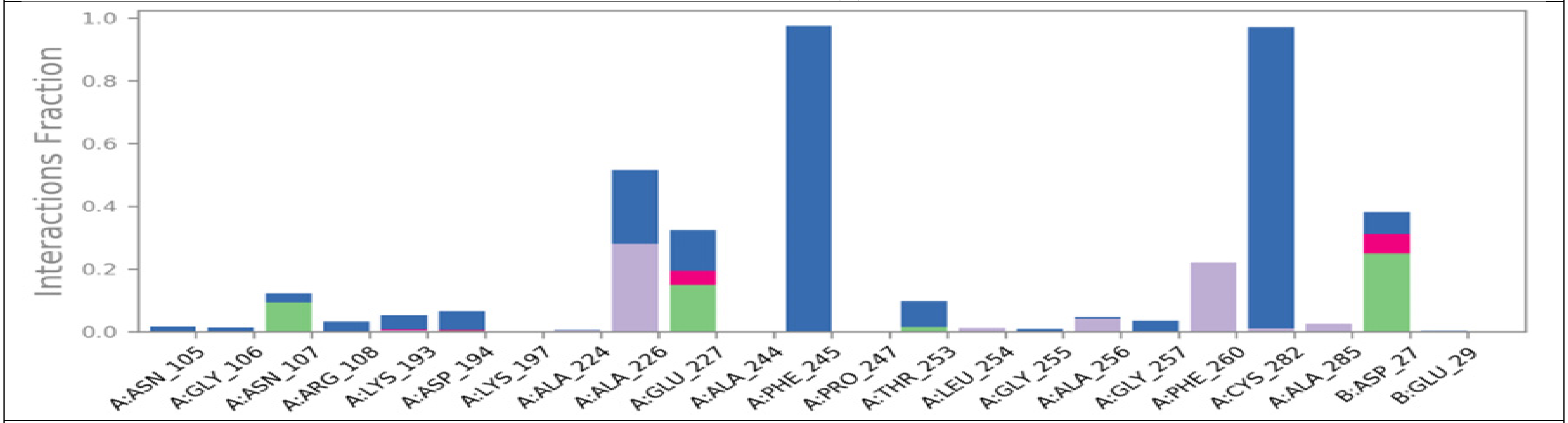

Co-crystal ligand (D)

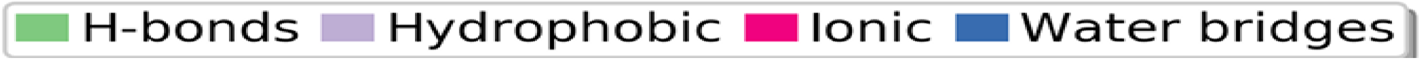

Supplementary Figure S2. Histogram of interactions for Hit 8 (A), γ-Mangostin (B), LY-3522348 (C), and the co-crystal ligand (D). The figure compares the interaction profiles of the compounds, highlighting the distinctive hydrogen bond, hydrophobic, and water-bridge contacts.
